# Supplementary material for: Two complementary genes in a presence-absence variation contribute to indica-japonica reproductive isolation in rice
Source: Nat Commun. 2023 Jul 28;14:4531. doi: 10.1038/s41467-023-40189-x (PMC10382596; doi:10.1038/s41467-023-40189-x)
Supplement: Supplementary file 1 — Supplementary Information [file 41467_2023_40189_MOESM1_ESM.pdf]

Two complementary genes in a presence-absence variation contribute to *indica-japonica* reproductive isolation in rice

Wang *et al*.

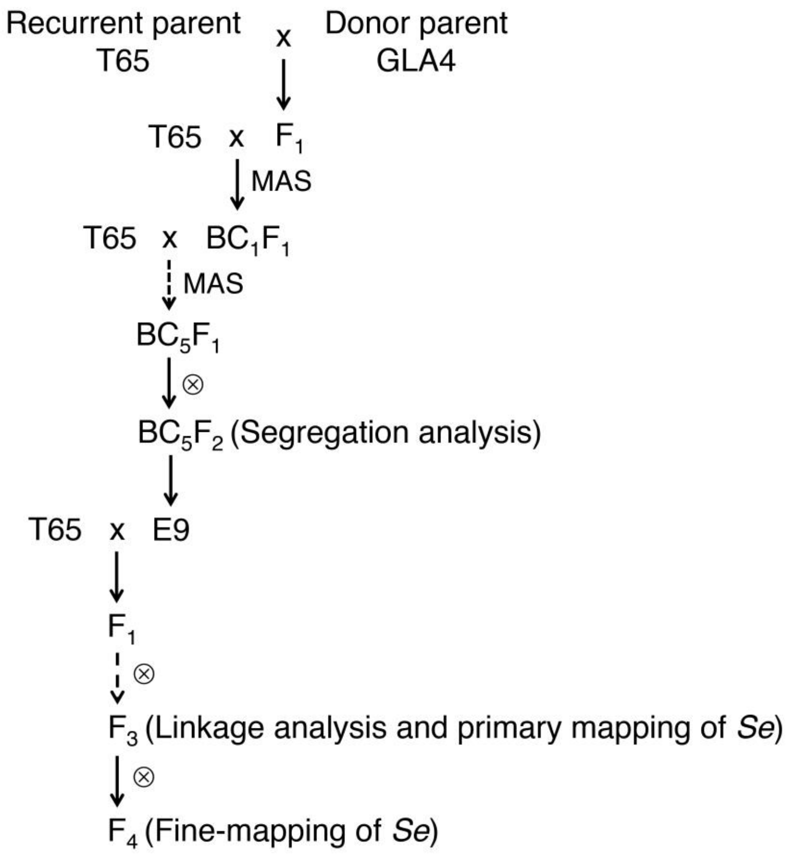

Supplementary Figure 1. Flowchart of the development and analysis of E9.

MAS: marker-assisted selection.

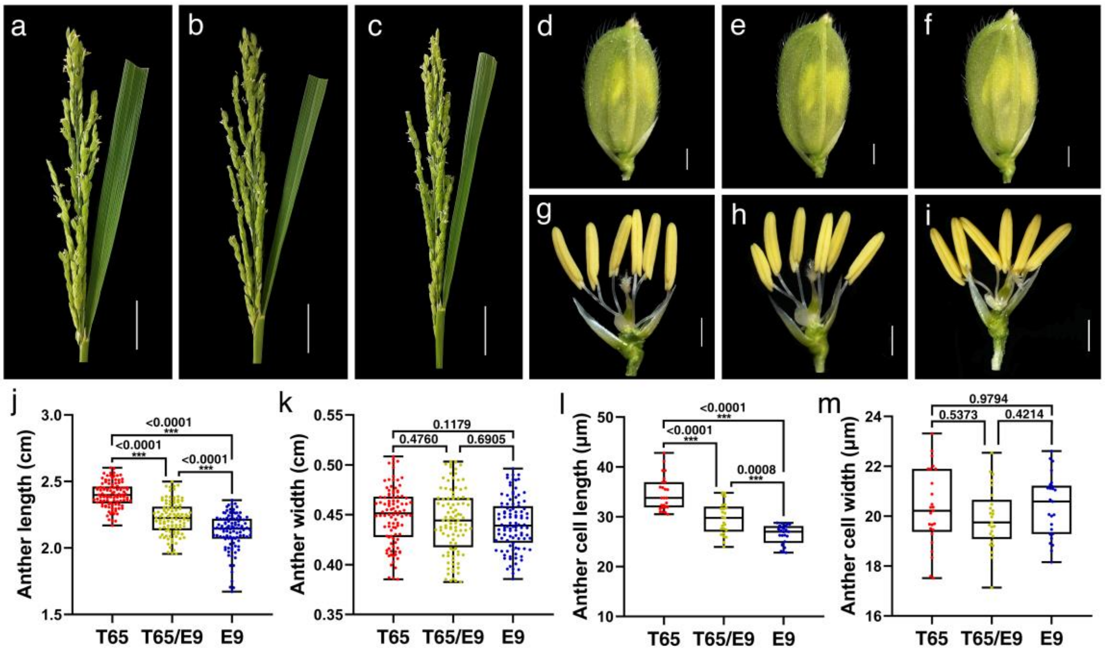

Supplementary Figure 2. Comparison of florescence-related traits of T65, E9 and T65/E9 F_1 hybrid.

(a-c) Panicles of T65 (a), E9 (b) and T65/E9 F_1 hybrid (c) at the heading stage. **(d-f)** Spikelets of T65 (d), E9 (e) and T65/E9 F_1 hybrid (f). **(g-i)** Spikelets of T65 (g), E9 (h) and T65/E9 F_1 hybrid (i) after removing the lemma and palea. **(j-m)** Comparison of anther length (j), anther width (k), anther cell length (l), and anther cell width (m) among three genotypes. Data are shown as means ± SD (n = 100 and 25 biologically independent samples for j-k and l-m, respectively). Scale bars = 3 cm in a-c, 2 mm in d-f, and 1 mm in g-h. Two-tailed Student's t-tests were performed to determine significant differences (***p<0.001). Source data are provided as a Source Data file.

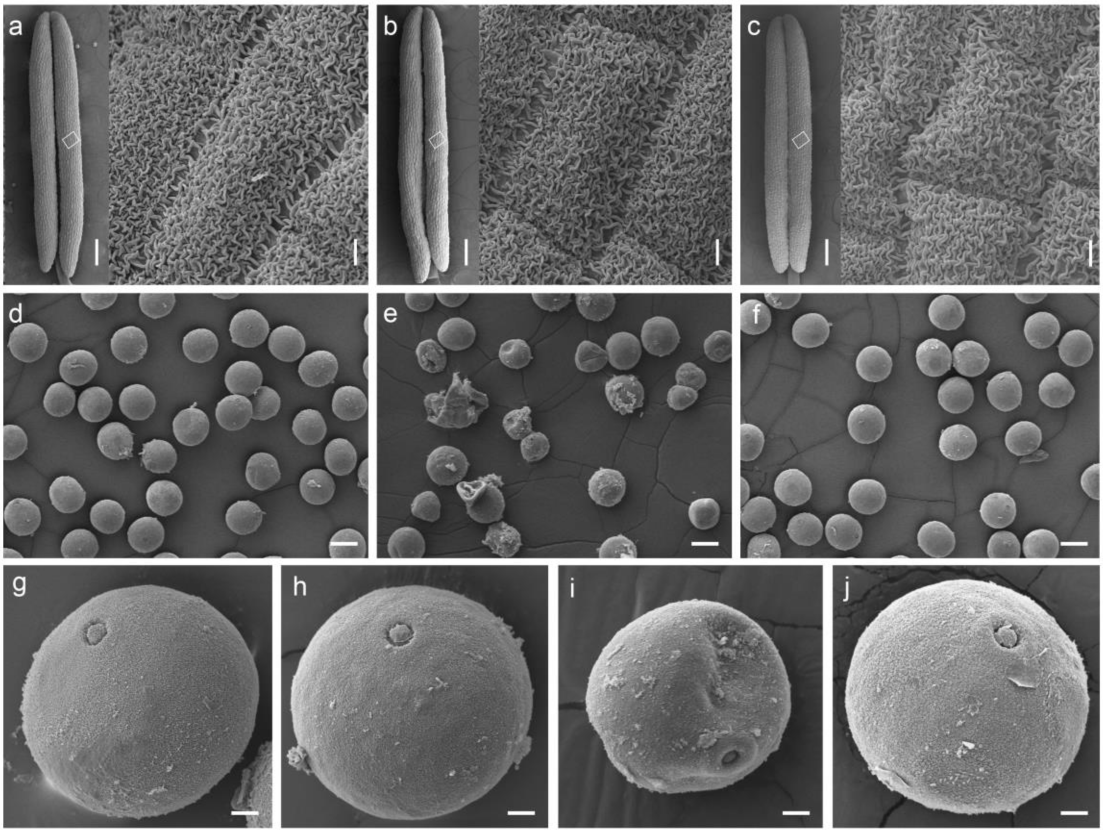

Supplementary Figure 3. Scanning electron microscopy (SEM) examination of anther and pollen.

(a-c) SEM of anther (left) and anther epidermis (right) from T65 (a), T65/E9 F_1 hybrid (b) and E9 (c). **(d-f)** SEM of mature pollen grains from T65 (d), T65/E9 F_1 hybrid (e) and E9 (f). Mis-shaped and presumably sterile pollen grains were observed in T65/E9 F_1 hybrids. **(g-h)** Representative pollen grains from T65 (g) and E9 (h). **(i-j)** Representative samples of defective (i) and normal (j) pollen grains from T65/E9 F_1 hybrids. Scale bars = 100 μm in a-c (left), 2 μm in a-c (right), 20 μm in d-f, and 2 μm in g-j. *n* = 3 independent experiments.

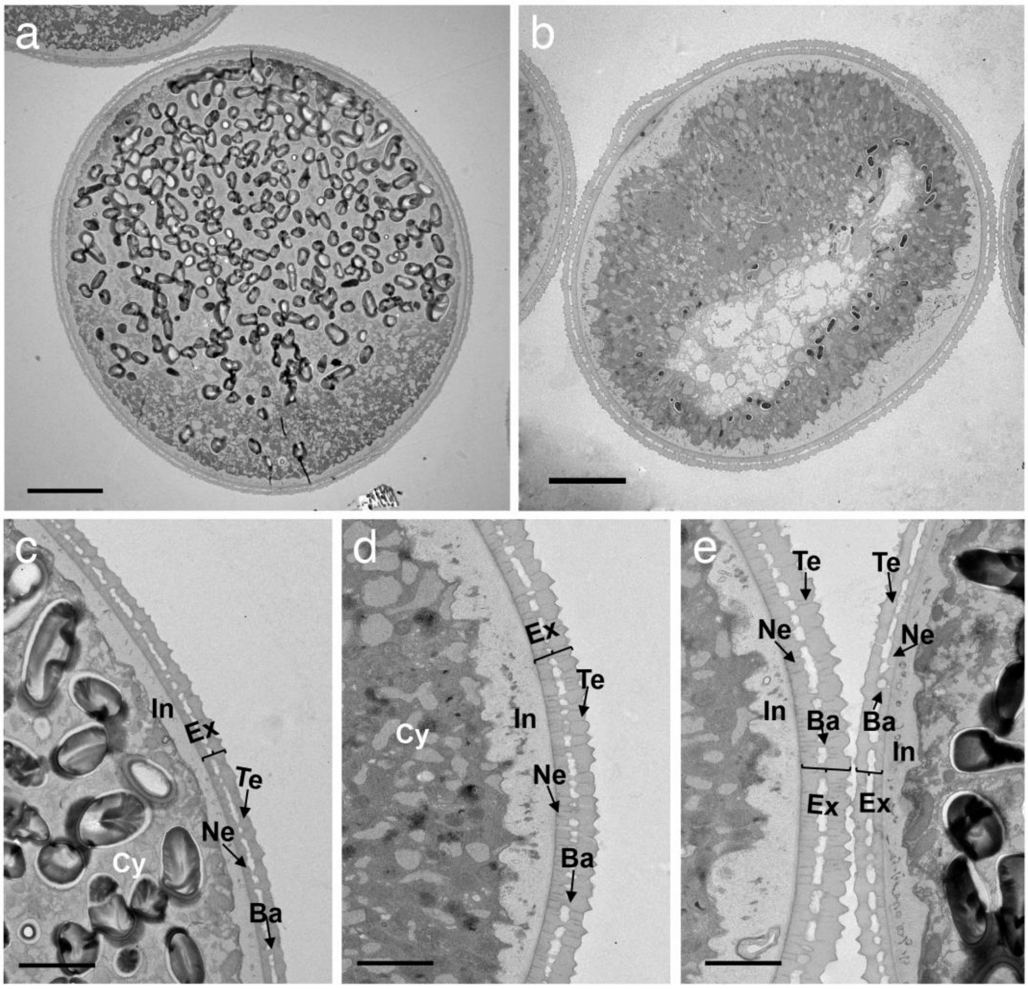

Supplementary Figure 4. Transmission electron microscopy (TEM) of pollen from T65/E9 F_1 hybrids.

(a-b) TEM of normal (a) and defective (b) pollen grain from a T65/E9 F_1 hybrid. **(c-d)** Higher magnification images of the pollen grains shown in a (c) or b (d). **(e)** Comparison of the pollen walls in defective (left) and normal (right) pollen grains in a T65/E9 F_1 hybrid. Ex, exine; Te, tectum; Ne, nexine; Ba, bacula; In, intine; Cy, cytosol; C, cuticle; CW, cell wall; Ep, epidermis. Scale bars = 5 μm in a-b, and 2 μm in c-e. *n* = 3 independent experiments.

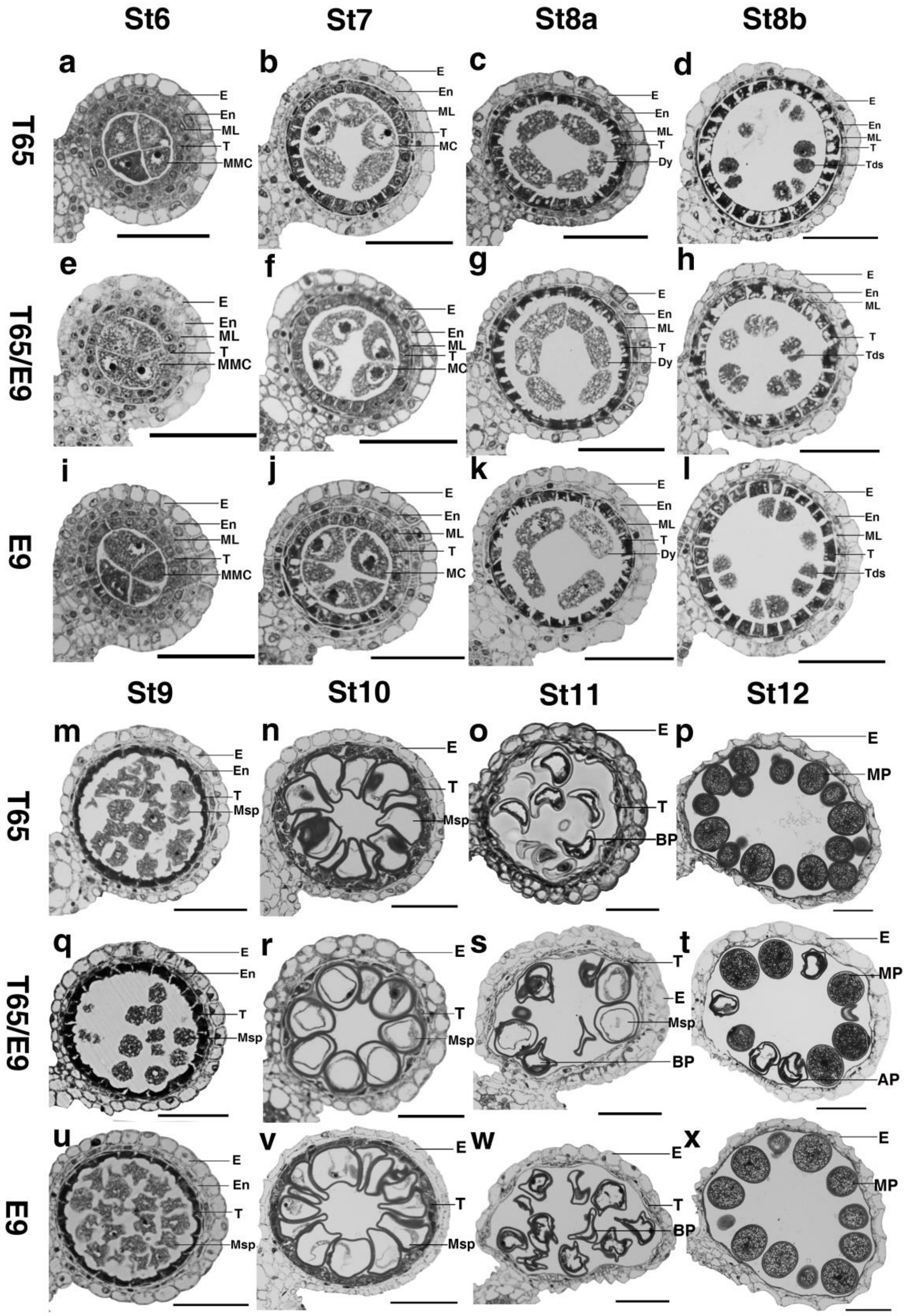

Supplementary Figure 5. Semi-thin section comparison of anther development of T65, E9 and T65/E9 F$_1$ hybrid.

(a-n, **q-r**, **u-v)** No obvious differences in the development of anther were detected from meiocyte mother cell (MMC) stage to vacuolated microspore stage (St6 to St10)

among the three genotypes. (**o**, **s**, **w)** At bicellular pollen stage (St11), half of microspores were aborted in the T65/E9 F_1 hybrid (s) but not in T65 (o) or E9 (w). **(p**, **t**, **x)** At tricellular pollen stage (St12), a significant portion of aborted pollen appeared similar to immature St10 pollen in the T65/E9 F_1 hybrid (t), whereas mature pollen grains filled with densely-staining starch granules were observed in T65 (p) and E9 (x). St6, MMC stage; St7, meiosis prophase I stage; St8a, dyad stage; St8b, tetrad stage; St9, early microspore stage; St10, vacuolated microspore stage; St11, bicellular pollen stage; St12, tricellular pollen stage. E, epidermis; En, endothecium; ML, middle layer; T, tapetum; Dy, dyad cell; Tds, tetrads; Msp, microspore; BP, bicelullar pollen; MP, mature pollen; AP, abortive pollen. Scale bars = 50 μm. *n* = 3 independent experiments.

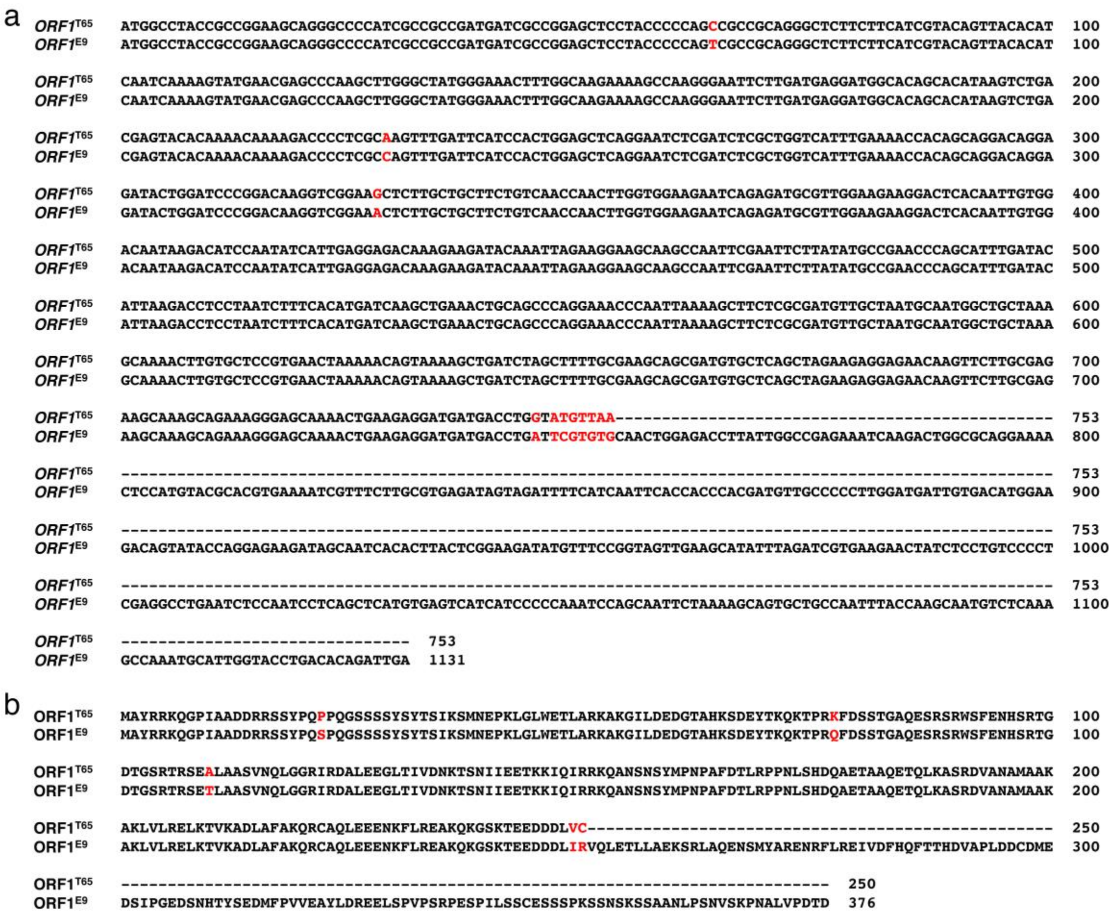

Supplementary Figure 6. Sequence alignment between *ORF1*^{T65} and *ORF1*^{E9}.

(a) Alignment of coding sequences of *ORF1*^{T65} and *ORF1*^{E9}. **(b)** Alignment of protein sequences of ORF1^{T65} and ORF1^{E9}. The nucleotide substitutions and amino acid differences between T65 and E9 are shown in red.

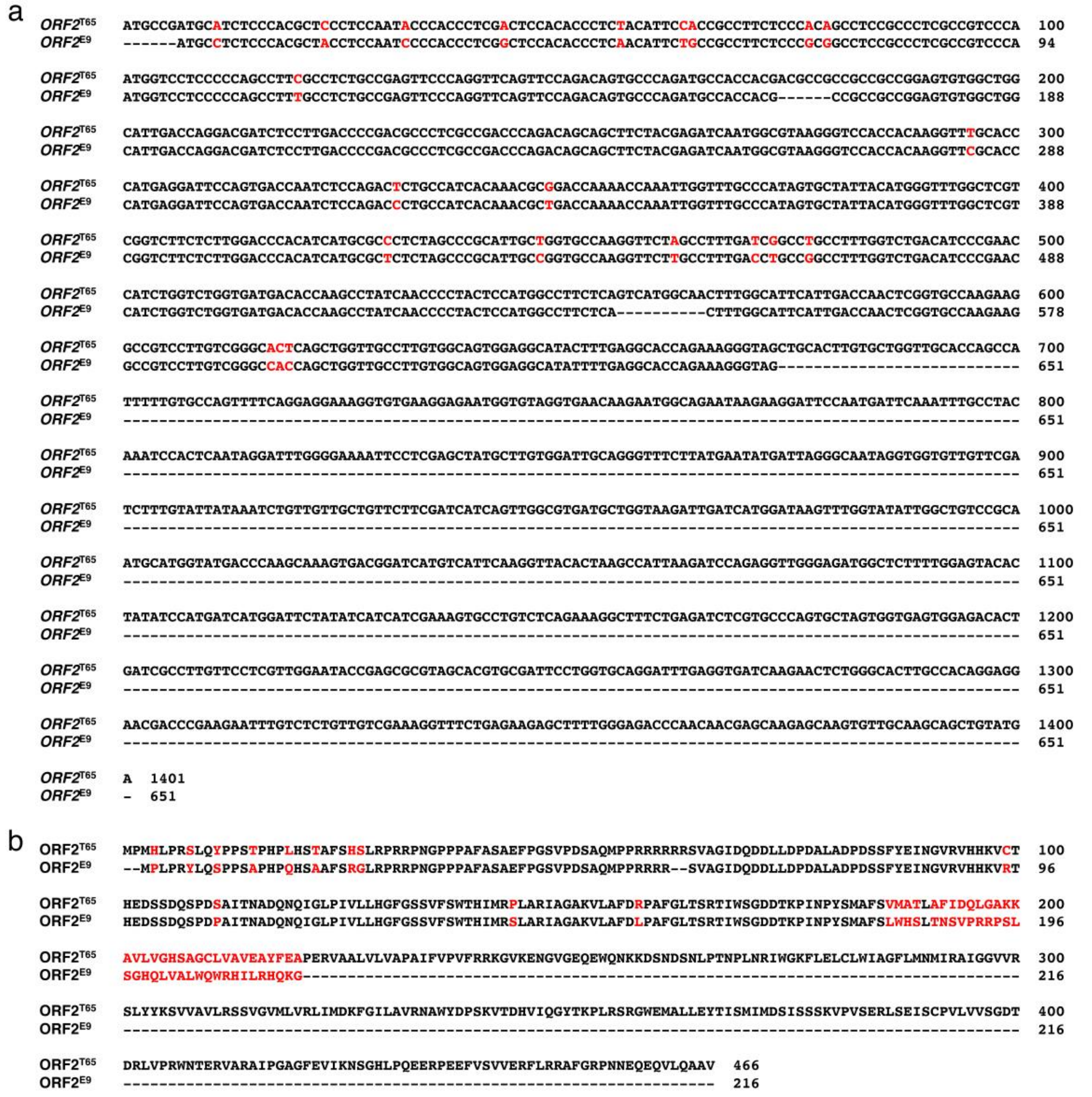

Supplementary Figure 7. Sequence alignment between *ORF2*^{T65} and *ORF2*^{E9}.

(a) Alignment of coding sequences of *ORF2*^{T65} and putative *ORF2*^{E9}. **(b)** Alignment of protein sequences of ORF2^{T65} and putative ORF2^{E9}. The nucleotide substitutions and amino acid differences between T65 and E9 are shown in red.

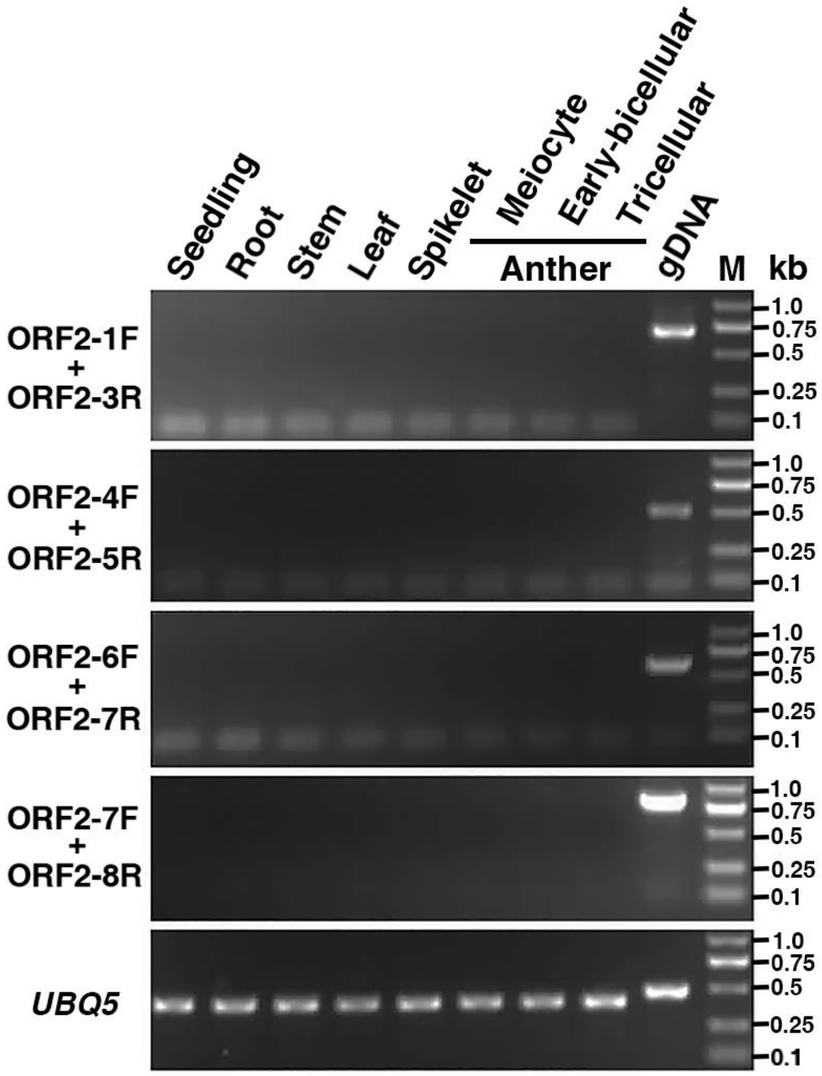

Supplementary Figure 8. Expression analysis of *ORF2*$^{\mathrm{E9}}$ in different tissues and different stages of anther development.

Four pairs of primers in different locations of putative *ORF2*^{E9} were used to test its expression in E9 plants. *UBQ5* was used as a positive control. n = 3 independent experiments. All primers are listed in Supplementary Data 4. Source data are provided as a Source Data file.

a

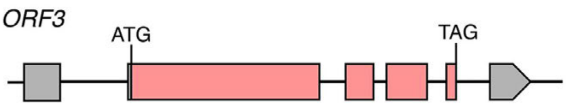

GCATCCCTCAACCCTAGCTCCCCACCTCTCCTCCTCCGGCGGCGCGGAGGGCCGAGAGGGAGGCGGAGCCTGGCGTCCCAGGGGGT
GGCGCGGGAGGCGGTGTCCGGCGGTGCGGGAGGCGGAGTGCGGGGGCAGCGCAGGAGTCTGAAGGCGGCGTCGTGTCTGCATCAGC
ATCGCTTTTCTCCTCCGAATCCTCTCCCAAAAAACCGAGGTATTCTCTCAAAACTTAGGTTAATTGTTTTTGGGATTGATTCGTTC
AGATCTGGTGTATGTGTGCTTGTTTGTGCCACCATCTAAGCACACCCTTAGGCTTCGTGGTGAGGCACACCCCCTACCCTCTCTCT
TTCTCTCTTTGTCTGATTTGTTAATTTCTGGATGATTGGATAAATGCTTGCAGCCTCTCATGGCTTAGATTAGAGCACTGATAGAA
ATGAGTTACATGATGATGACAATTATGATTGATTGATTGCAGTATATGGATAATACGATGAGGAGTTTAGTTGATACTGAATGCTG
CACATATCATTTCTGTTAATTACAAAGAAGCTTGTCATTGCCCCTGTTATCAATTTATTCATCATGTGGCATGTGGCATGTGCACT
AATAGGGCTTTCTTTAAAAACAATGTGCACTAATAGACAGCATCTCACAACATTGTCTTCAAAGTCTGTAGCAAGAAAATTTCTTT
ACGATATCTCTAATTTTTATCTGGAACAATTATTTTTTGTCAGCAGAGAAAT**ATG**TTGGTAACCAGATCGAGGTTATTTGGAGTGT
CCCAGTTGCGCAAATCCTCTCTTAAGCATCTGTTGAAGTTCAGGTCTATGTCTGATCAGTCATCTGTTCTAGCTGCCGATGATACC
GAAACTTCATGGGCTATGTCTGATCAGTCGTCCGTTCCAGCTGGCAAGGCTACTGAATTTTCATTAGGTGATTTCCCTAAGCATCT
GCGCAAGCTACCACCATGGATCCAAGAGCCGGCGTCTGTTCATGAAGTTAATGATACCTTGAAGCACATTGAAGAAAAATCAAACG
AATTCAAGCTGCTTTCTTCGGATTGCATGCAGAAGAAAATCTTTGAGCAAGAGATTAGACCTTTCTTACTCTCAAGAGGTCTACTG
TGCACTAGTCCTATGGGAAGCTCGTTTGTCAAAGGTTTGCACAAGATGAATCATACAATTCATCACAAGTTTCCACGATATGTCAC
CTCTAAGAGCTTGATGAGGCTTCTGACTACACGTAGTCAGAAACAATGGAGACCAATACTCGAGTATCTGGCCGAGGAGCGAGTTA
TAGTTCTCTTGGACTGGATTAATGAGCTATCACTTCTTGAAGTACATTTTTTTGACCATGGGTTGCTTTTGGCACCACAGTCTGGA
GAGCCACATGATGCTCGTGTGTACAAGCTTGAGACTGATGCATGTTACCACAAGCCTGAGGCATCAAGTGATAAGAAGAAAGATAA
ATTGAAGAAATCAGTAGTGCCAGTAACCAAGTTATCTTCCCCTGAGAATGAAGAGCAGACATCGGATCCTCTTGGTAGAGCTACTG
TTGCGTCTGTCCTTTGGGAGTCCATCCAGGCACGCACAAAGTTAATTGAAATCCAGATTGTTAAGGGACTGTTTTGCAAAGATTCG
GAACACGCTGAAGGTGTTGGACTCTTCATGGCCGTTAAAAGGCCTAAAGAACTTGGGATAAGGAAATTGGATGTGGTCACTGACAA
TGAAGCAAACTACAATGTTATGATCGGCAAAAGGGATTTGAGCAAGAGTAGCAGTCCTGAAACCTCGATGGCATTTATCCAAGCTG
CGAAGGAATACGACATCTGCAAGTGCAGGAGAGAGCCAAGGGAAATGATAAGCCATGTGAATGACCTCGCCAAGGAAGCACACACC
AAAAGCACTGACATGTTGGATCTTAGAGAGATCATTGAGCGCAAGTCTCCTCAATTTTGGGGAATGCCATTTGTGCGCATCAAGCA
AGATGATTCTTCTATAAAAGCAAAATTAGGTAATACTCTCTCCATTCACATTTGATCAACGTATTAGTGTGAATGAAATTAGGATG
AGGGCTTCTAGGAGATCCTCCAGTTAGTGTGATGTTTTTTTTTTTGTCTTCTTTATTGCACTTTACACTAACTTATTTTTAATGTAG
GAGACTCGAAGGTCAGCGACACACCTTTTGAAGCAACTAATTATTACATGGAGGTCTGGAGCAAGGCTCAGAAAAATGATGCTTTT
GAAGGGCTATTGCGTGACCTTGATCCTGACTTTGTGATAGTATTTGCAAATGATGTCTCTGCTGAAGAGGTTATACTCGAACTAAT
GGCCTTGATTCTGTTTTGTAACTTTTTGCATCTAACGTGTCAACACTGCAGGTTATTTCAGAATTAACGTCGTATGATTACCCTTC
CTGTATTTGGGAATCTGGAGCCAATGTTTCCCAGCATTCTATTCCTGACCACAGAGCACACGTACTTATCAATCACAAGGGTTGCA
TTCCGCATTTTACCGGTAGCAAGTGCTTGATCGTTGCTTATGACAGTCCACCAGAGTTGATCCAGTATGAAAACAATCAAGATTCT
TCAGGAGTTATGTCTGTGGAATTTGTTGGACCTGGAAGTAAGCCACATTTGCATACTGACACAACCAAAGAAGTGGACCCATTCCT
TTCTTTATTTTTCCATCACCCTGGTAAATTGCAGTTACTACCTTTGCTATTTGTAAGAGAATAGCTGCTTTAATTTTTTTTTTCCT
ATGTCAAGAATAAACAAGTGCATGCAAATTTCTGCAGGCCGCAAAGGAGTAGAAGGTTGTAGTGGTGTCGAAAGTGGCAAGAAAAA
A**TAG**GTAATATTCTATTTTATTGGGTTATCCTGGTTGCTCTCATTTTCGTATCAGTGCTGCCTACTAGATCTTTTTGAGTTGGTTT
TGATTAAATTGTCACTGTTGTAAGGGCTTGCTAAGCAGTCCTGTCTCTTAAACCGCTCCATGTTCATCTCCCCCATAATTAACTCT
CTGCTAAATATGATTGTCTTTTAGTGTAATGCTTTGCAGAACAAAATACTCTGTGCTAAATATGATTGTCTCTTAATGTAATGCTT
TGCAGAGCAAGATTTGAGCTTTAAAAGATGGAGCCAGATTGAAAGAGTGCAAACCTACTTTAATGGGCATGGTTTTGGGAGAGCTG
TCTTAGGTTTTGAAGACAAGGACATGCTGTTTTTGGATGAATTGAACCGGATGGCCTTACGCATAGTTTCACTACTGACTTATGTT
AGAGGAACAGGAACTGTAAGATGTAGACAGGCAGTAAAATTTCAGTCCGACTACTGACTTCGGCAATTCTATGCCAAAGATTAACA
CCTTGGGAAATATCATGCACTTGTTACAGGCTCA

b

MLVTRSRLFGVSQLRKSSLKHLLKFRSMSDQSSVLAADDTETSWAMSDQSSVPAGKATEFSLGDFPKHLRKLPPWIQEPA 80
SVHEVNDTLKHIEEKSNEFKLLSSDCMQKKIFEQEIRPFLLSRGLLCTSPMGSSFVKGLHKMNHTIHHKFPRYVTSKSLM 160
RLLTTRSQKQWRPILEYLAEERVIVLLDWINELSLLEVHFFDHGLLLAPQSGEPHDARVYKLETDACYHKPEASSDKKKD 240
KLKKSVVPVTKLSSPENEEQTSDPLGRATVASVLWESIQARTKLIEIQIVKGLFCKDSEHAEGVGLFMAVKRPKELGIRK 320
LDVVTDNEANYNVMIGKRDLSKSSSPETSMAFIQAAKEYDICKCRREPREMISHVNDLAKEAHTKSTDMLDLREIIERKS 400
PQFWGMPFVRIKQDDSSIKAKLGDSKVSDTPFEATNYYMEVWSKAQKNDAFEGLLRDLDPDFVIVFANDVSAEEVISELT 480
SYDYPSCIWESGANVSQHSIPDHRAHVLINHKGCIPHFTGSKCLIVAYDSPPELIQYENNQDSSGVMSVEFVGPGSKPHL 560
HTDTTKEVDPFLSLFFHHPGRKGVEGCSGVESGKKK 596

Supplementary Figure 9. Genomic structure and protein sequence of *ORF3*.

(a) Genomic structure of *ORF3*. Exon sequences are shown with blue letters. Start and stop codons are underlined. **(b)** Protein sequence of ORF3. The mitochondrial targeting peptide (residues 1-30) predicted by iPSORT (https://ipsort.hgc.jp/index.html) is indicated with underlined black letters. A superfamily II DNA and RNA helicase domain predicted using the CDD tool (https://www.ncbi.nlm.nih.gov/cdd/) is indicated with underlined blue letters.

a

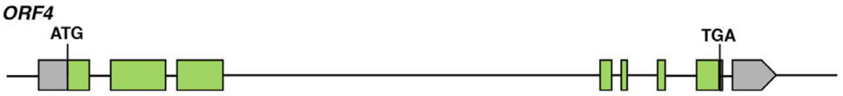


```
TTCCGTCGCCGCCGCCTACCCTCGACTACTGATCTACCTTTCTCCGTCGGCGGCCGCGGGTGCCCAGCGCAACCGCACCCTCTCCTCTCCAGCAGCGGCGACCCCT
CGCCTCATACCCTTTCCTGGGCAAGGCCGGGAGGGGATCAATCTAGGGTTTCCGCCGCCGCCGGTTCCCCGGACGTTGCTGCATGACGATGGAAGATGGAAGCTGT
CCTTCACCAGTCACCCCCAGCGAAGCCGAAGCTGGAGCCGAAGATTACGAATCTTGGACACTGAAGCAGAAGATTGAGGATCTCGTCAACTGTGATCCTATCCATG
GTATCATGCCAAAAAATCCTAAATATAAAGCATATTTCGAGGAAAAGTTCCAGGTACGTACGTACATATTTTCTTCGATCCATCATCCATGGATGCAAAACTAATT
AATTAATTTTGACCTCACCAGCTAGCATATATTTTTTTTTATTGTATGCAATCGATCGATGAATCAAGTGGTATCATATCTATTGCTATTGTAGGAGAAGTTAAGT
AAGTACGTCCGTGTTGTATTGCCGAAACTTCGTCCAGCAATTCAGAAGGATAGCGTCAAGCAATTCTTTCAAGTCTACAATTGTTGGTCAGGTTTCATGGGACGGG
GAAACTTCCTTTTACCTGATATCCTCACCAACATGGCCAACAAGAACGCTCTGCGTTGTGCCAGGGTCGCACTCCAGGGCACCTCTCCTCTGTTGCGTCGCCGCCG
CGCTGACCCCAACACCCGCCACCGCTATGGCTTCGCCCCCCTACACATGGCTGCCGAGAACTTCAGCGTCGACATGGTCAAACTCCTCTTCCGTTACGGCGCATCG
GCGAACATCCGCACCAAGGGCGAGTATGTCATTGAGGGCCTCCTCCCTCTCCATGTTGCTGTCGAGAATGCCAGCATGCACAAGTACATTGAGGATCATTGGGCTC
ATGGGGACCATATCATCAACCTCATCTTCCTCCTTTGTCTTCCTGAGATGGTTTCTTCTTCATTCAATTCCTATCCCTATTATATTACTGCCAGAGCATTTAGCCT
ATTTCAAATACTAGCTTGGACCAATAATTAATTTCAGCCACCACTGACATGCAGAAAATGTTCTTGGACACGACTAGATTAATTGCCAAACAGACAGATAACATTG
TCGACGAGGTATGGAATTACATTCGTGAAGAAAAGCATGTCCAGGCAGCAATTTTGCTGCTGGCTGCTCAAAAGCAGCTCCGTGGCCGCCTTAACAACAGCAGTGG
CAAAGCCTCTCTGAACGGGTTTGATATTGTTAAAAGCCACATTGATGATGCTCTTAACACCATACATCTTGAGGGGTTAAATATGGTTAAGGAGGGAAAGAATGGC
AGGGCACTAAAGAGATTGAAAGACAAGAAGGAAGCCCTTCTTACAGCACTTGTACTAGTTGGCATTGTTCACAAAGCTGGTGAAGCTCTTGAGGGATACATTCAAA
CTCATTCACAGGTAATTGCTGAAATTTCCCCATTGTTACCATTGCTTTCTGATACCTGTGGGTTTTGATTTTTCCAGTTTCGTTTTCTTTAAATTTGTGTGATGCT
TACTTCCCATGATTTTGATCTACTAGTGACTCATTTTCTTTAAATTTGTGTGATGCTTACTTCCATGATTTTGATATACTAGTGAAACCTCGAATTACCCTTGTTT
TAGAAAGCTGTGGTGGGTGTGGATCGCTTTTCTTTTTCTTTTTGGTGATATGTAAACAGTTGGAGGAGCTCCTGCTGCATTGTATCAACTAGTCACATCCATATTA
TAAAAAGGAGTTAAAAAGAACTGGACCCATGCAGTTAATGGCATCTTTTAGCCTCCATTTTCATGTAAATCCAAGAGGCTGAATAGCAGGCCCAGCATAGTGGTAG
TATACTGCTAGGTTCAGGTCTAATGCATGAGTCAAAAATATATGGAAACATGGGAGTAGATGCAGTGTGTTCTTAATGCGAAGCAAGGTGGCCCACCATCTTTTAG
ATGCCTACGAGTTTAATGCATAAGTCAAGGTGACACCTTACATCTAAATCTATACAATGTTGCCAACTTCTGTATTACACACATGCTTACCAATTATTGAGCCCAT
GTACCATTCGTTTCCCCTTTCTAAAGAAGATCTAAGAGGAGGGGAAGAGGTCCTCTTCAGTCTCAGCAGCAGAATGCAAGAAGGGAAAAAAGGACATGTATCCTGT
CCAAAGAGGCAGAGCAAAGCGCAAGAAAAAGAACATAGATGAGGGAAGAGGTGCCCTTTCCAAACTAGCAGAGCAAAGCATAGAACAAGAAACAGAGCAGAGAAGA
GGGAAGAACAAGTACCTGTCCTGTGCGACGGTTGACAGCTGGGGAAGTGGAGAGCATGAAGGAGAAGCAGACCCGTCGGAGAAATAGGGGCGTTAGGAAGGCAGGC
TGGTCCTCTGCATCTCCCCCTTCTCCTTTTCTATCTCTAGGCCACTAGCACCCCTCACACACCAGCCTTTCGACTGTGCACGCCACACTTGCTCTCCCCACATGTG
CGTACCCCCATGGCACTTGCGGAAGGCTAAGCGAGCAATATGCTCTCTTCCACTGGTGCTTGGGCGCGAAAGGCGATGCCTTTACGACACTTTAAAAACCAAGAAT
TGATATTTGCATGAACTATAGAAGAGCATAGGGGTCCATTGAAGGTAAGTTTATCTTTTTGGGATCAGGAACATATAAAATTTGTAGGGAAGATCAATTGTTCTTA
AGTATGGTTTGAGAATAAAAATATAATAGCACCCTCCCTTGGATTGGGCTACCTTTTAAAGTGGGACCCCATGTAAGTTTAGCAAAGCAAGTTTTTTAGCTTGGAA
ACTGAGTCTTTGAGTTTTTGTAAATATTTTCAATTTTATTCATGAATATATGCGTATTCAGCAATTTTAATTATGACTCCATATATATTCAGCTTCTATTCTTTTT
TTTTTTAAGTTGAGGGCTAACATAAAGGGTAAGATGAGCAAGAAGGTGCGTAGTTGTTATGTGGGAGATCATGAGGCTAATAAAGGGAATAGCACTTAGATTATG
TCAAGGTAACCTGCAAAAGGGTAGAGATGAATAGATGATAGTGTTAAGGGTGATGGTGGTTTGTCTTTAGCCCCATTGGAGTGGAAACCTGATATTGGATATGCTG
CCCTTGTTAATAATATATGTTTGAGTGGAAGTTTATATATTCTTTCTCTAACCATGACTATGACTAGGAATCATCATATTGTTCTCTCAACAAGTCTTTCATATTT
TAATTGAATTCAAGCTCTAAGAAATGAACGAAACAAAAGCTGGACTACCCTGAAGCAACTTCTCATAATGACCCAAATTACATGACCAGAAGGCCTGCATTATGGA
AGCTTTTGTTGGTGCTGACCAGTTCTTTTTCTTCCTCATTTATTTCTTCTATTCCTCACCTGGCATGTTCATGTAAAACGCAAAACACATGTCGGATTGTTATTTT
CTTTCTTATTTATTTGCTTTATTCTACGTGCATGTTAAAGTTTAGTGAGAATGCATGTGTCAAAAATTGTGACCCTCTTTCACCCCACGTCTCTATGCACACGAAG
AGTGGGTTCTAATATTTTCTGTTCAACTTTACTTGGGTCTGTTTCGAAGGCCCGCCATTTCACAGCATCCATATATGCCATCATGCCACAATGAATATATCAATAA
ATTTAAAAAGTGGATGCTTGAAAAGAAAGATTAACCTGAACAATCATGGTATTACCATCATGCCACAACTTAGTTGGCAGTGACGATACATGTTCACATGGTACCT
ATCATAGGAGAATAATAGTTTAAGCGTTAAGCCTCCACCTTCTCACATCAAGCCTAGCAGAGAAAAGGCCTTGGCTTTTGGTGTCACTTAAGATTCGTTTATCATT
AGGCAACCTTCTATAGTTTGTCTTTACTACTTTTTTATTTGTAGTAGGAAAAGTGAAAAATTCTCTGCTGGGGTCCATGGCAATGAAACTGCAAATTCCATGCAAC
TATCTATACCCCCAGAGTGTAGAACATAACATTGTGACACCACAATATTCTAGAGTTGATTACCATAGAGCATCTGTATAGTTTAGACTGTTATACTTTCAATTCC
AAAAAAGTGATCTGGTTTGTAGCAGGTGCAACGGTACCGAAAACAACTCTCACCTGCCAATATTCTGCTTTACACGTGTGCCGGCATAGTGCTGCCATACCACTGT
TCTGTTGTGTAAGATCTGTTTTAATTTTCTTCTGCTGTTATACTGTACTGTCGTCAAGTAATGTGCCATTGGTTCCAGACAAAAAGTATAAGTTGGGCAACTTGGT
TGTTTGTTCATCTAGCCAAATGAGATGTTATTGACAAGGCCCTAGTGGGCTTTTTGGTGTTGTCTTGTTTGCGGTGAGCAAGGATGGCAAATAAATCCAAGCTTGG
AATAGTAGAGTTGGTTGACTATGGCAGATTGGCAGCATGCCAAATCAATGACTTACTGATTCAACTCTTGACCACTTAGTATTTGTGTAGATGTAGTGCTTAAAAC
ACCATTGTTGACTTTCAGATGGTCTGTACTGTTTGTCTCGAGAGATGATGTTATTGAAGGCTGTTATGTGTCTTGCAACCACCACCACCTGCAAGTGTAGATATCT
GAATTCTCGTTTTGGAATAATATCCTTGAACATTTTGTAATGATACGTTTTAACAATTTTCTTGGTGAAATGTAGGTGCGCCATGACGAAATTGTTGAACATGTTT
CATCAATTCTAAAAAGCAATGGCATTGCTCATTCTGGGGAAAGCATTGACACTGGAAAACTTGAATGGTTTGACATCTACTGGCTTTATATATATATTCAGTTCAT
GTTTTTGTGCATATAATTTATGCGTTGATTTATTTGTGTTTTGAAGCTACCAACATGGTGGGGGATGTCGATTGGCAAGTCAGATTCACAAAGAGGTGTGTTTCA
TTTTGGTTGATCCTATTTCTTATTAAAATAAACTAGAAAGGAAAATAGCTGTGAAACTTTATTACGTGTCCACCCATTACAAAGTCATTTGCTGTGGTGAATGCAC
AACTTTGGAACATTGCAGAAAGCTTATGACTTGCATCCAGCGCAAAAGGGGGTGCAACCAGACTCTGTACACCAACCTCGGAACTATGCTTTCCATTTCATAAGCT
GTCTCATGACCAATTTCATTGCTGATTACAGTGGGCTATGGGGAAACTATTGAAGCGGACAAGTCATCTTCTGATATAGGCGAAGTTAGTAAAATGGTAATGAGAT
ACGCAATTAAATTTATTTGTTGAGTCTAATTCATGCTGATGAAATTAGTATGAAAACAAGATTGTTCCTCCAAATTTGCTCCTACTGACCCCTATCAATTAACAAG
ATATGGAAATATTTGATAACGTAATGCATAACCATCAATTGTTGCCTTATTGGTTAACAACAGAATGAATACTCACTACTTAAATCTGAGTGTTTTTATAATTTTG
TTTATGCGTTCAGTTGCTAATTTTCTCATGTAAACAGATCCTGGGAAAGCAACCACCTAAAGGATCAGCCATACGCGAAGTGAGAGATATGTTCTTCCCATACTGG
AAATCGGTGTTGTCTCGACGGTTGCAGCTAAAGATAGTTCCATCCTGTCAACTAAGCAGGAAGGACTTGCTAAGTGCTGAAGCGAGCACGAAGGGGACCAAGAGTA
TTGACCACCCGTGTAACCCAATCAAATCAATGGGAAATCTTGGTTCGATGGGCTGGCCCCCATTGTCAAGTGAGTCCAGAAGGATGCTTTATACTGTTGCTTCAAT
GTCCCGGAAGGTGTTTAAGCGCACTTGACTACAGAAAATCTTTGGCGGTATGCTTGTTGAAACACTTTTTTTTTTCTATAATAAGCCTCTAGAATGTCTTTTGGTC
ATAGTGCCCCCTACATTCTCAATGTTGCTTTAATCTTTGTGTAGGTCTCGGAAAGATCAATGAAACTTTGAGCCATCAGACGGTTGTATCCTATTGGGCTAGAAAC
TGTGTTTCATGGTTGTTATGGTCCATACTTTTTAATTTGAAAACCTTGATTGCAGTGTAATATTCAGATTTAAAATATGATGGAACGTCATTTATTTCTTGTAGTG
TGCCAGGCTAAAGCTGACCCGG
```

b

```
MTMEDGSCPSPVTPSEAEAGAEDYESWTLKQKIEDLVNCDPIHGIMPKNPKYKAYFEEKFQEKLSKYVRVVLPKLRPAIQKDSVKQFFQVYNCWSGFMGR   100
GNFLLPDILTNMANKNALRCARVALQGTSPLLRRRRADPNTRHRYGFAPLHMAAENFSVDMVKLLFRYGASANIRTKGEYVIEGLLPLHVAVENASMHKY   200
IEDHWAHGDHIINLIFLLCLPEMKMFLDTTRLIAKQTDNIVDEVWNYIREEKHVQAAILLLAAQKQLRGRLNNSSGKASLNGFDIVKSHIDDALNTIHLE   300
GLNMVKEGKNGRALKRLKDKKEALLTALVLVGIVHKAGEALEGYIQTHSQVRHDEIVEHVSSILKSNGIAHSGESIDTGKLECYQHGGGMSIGKSDSQRV   400
GYGETIEADKSSSDIGEVSKMILGKQPPKGSAIREVRDMFFPYWKSVLSRRLQLKIVPSCQLSRKDLLSAEASTKGTKSIDHPCNPIKSMGNLGSMGWPP   500
LSSESRRMLYTVASMSRKVFKRT   523
```

Supplementary Figure 10. Genomic structure and protein sequence of *ORF4*.

(a) Genomic structure of *ORF4*. Exon sequences are shown with blue letters. Start and stop codons are underlined. (b) Protein sequence of ORF4. A predicted ankyrin repeats domain predicted using the CDD tool is indicated with underlined blue letters. No mitochondrial targeting signal was predicted in ORF4 by iPSORT or MITOPROT (https://ihg.helmholtz-muenchen.de/ihg/mitoprot.html).

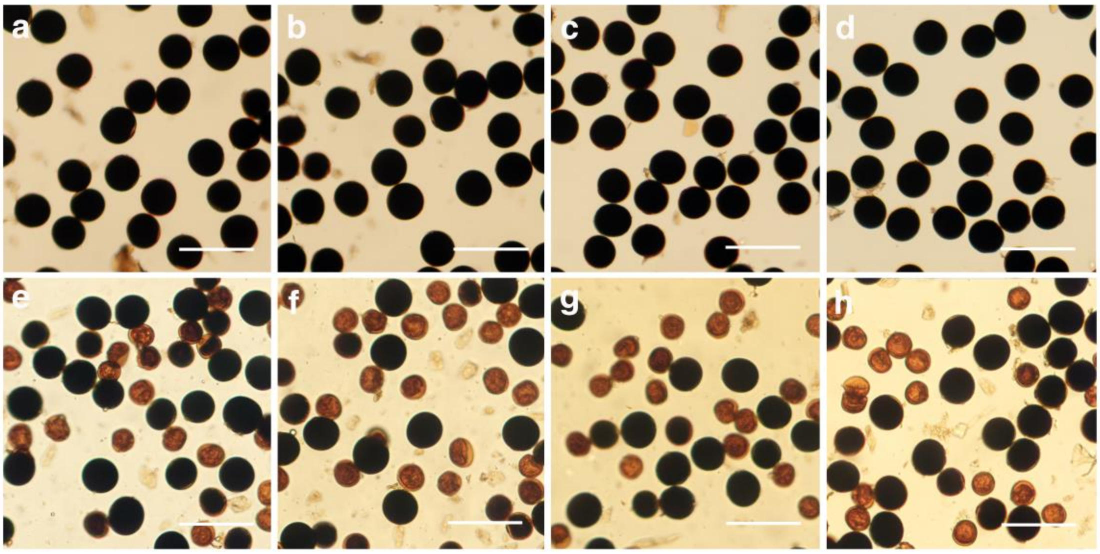

Supplementary Figure 11. Pollen fertility of CRISPR/Cas9-engineered mutants of *orf1* and *orf2*.

(a-d) I_2-KI stained pollen of *orf1*^{T65} (a), *orf2*^{T65} (b), *orf1*^{E9} (c) and *orf1*^{T65}*orf2*^{T65} (d). **(e-h)** I_2-KI stained pollen of F_1 hybrids of *orf1*^{T65}/E9 (e), *orf2*^{T65}/E9 (f), *orf1*^{E9}/T65 (g) and *orf1*^{T65}*orf2*^{T65}/E9 (h). Scale bars = 100 µm. n = 3 independent experiments.

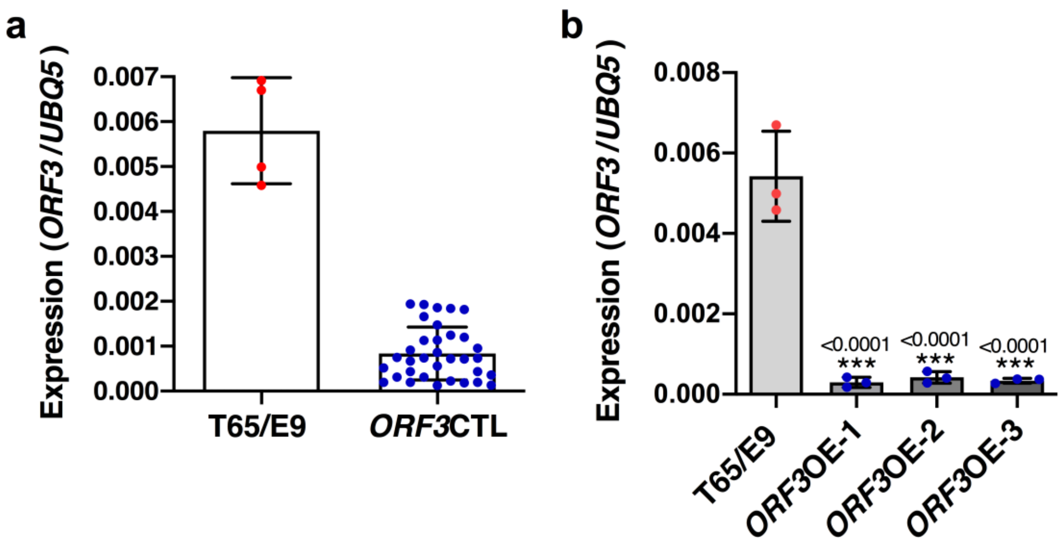

Supplementary Figure 12. Expression analysis of *ORF3* transgenic plants carrying *ORF3* complementation or overexpression constructs.

(a) Comparison of *ORF3* expression in 4 T65/E9 F_1 hybrids and 34 transgenic lines carrying a complementation (*ORF3*CTL) construct. Data are shown as means ± SD (n = 3 and 34 biologically independent samples for T65/E9 and *ORF3*CTL, respectively). **(b)** Comparison of *ORF3* expression in T65/E9 F_1 hybrids and 3 positive transgenic lines carrying an overexpression (*ORF3*OE) construct. Data are shown as means ± SD (n = 3 biologically independent experiments). Significant differences were determined by two-tailed Student's t-tests (***p<0.001 compared to T65/E9). Source data are provided as a Source Data file.

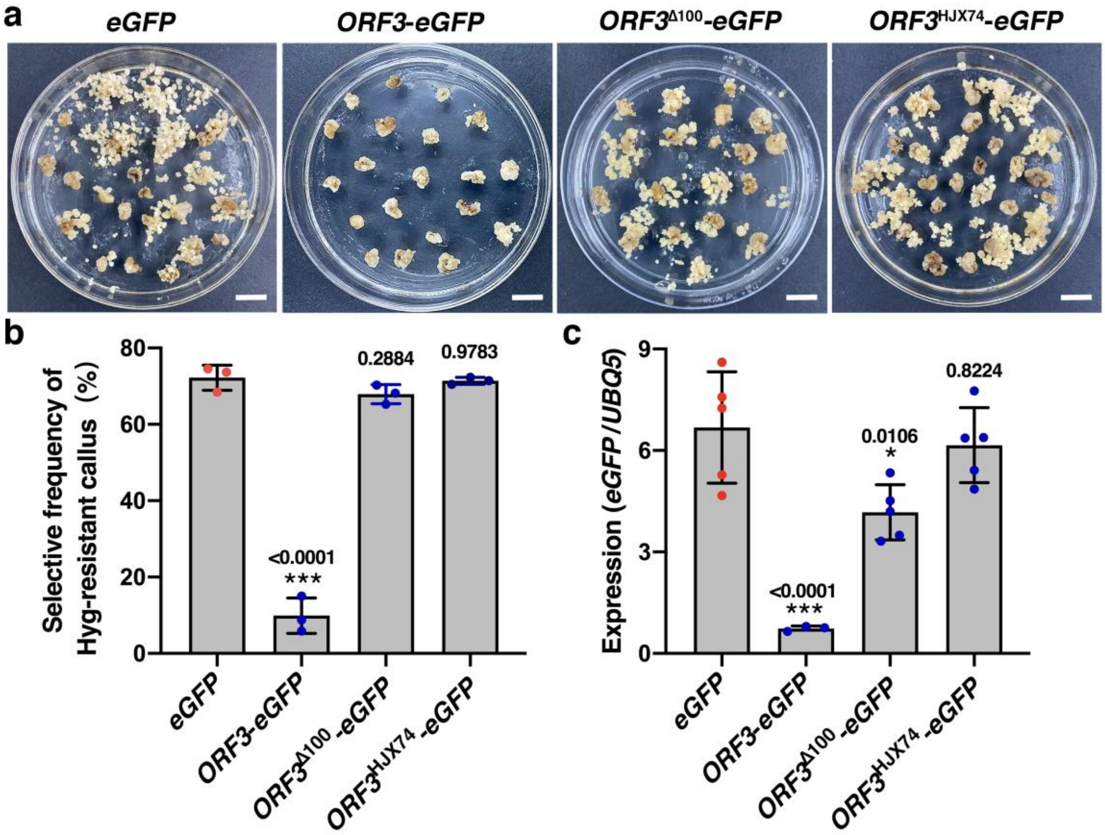

Supplementary Figure 13. *ORF3* influences genetic transformation efficiency under T65 genetic background.

(a) Transformed calli 30 days after the onset of selection. Hyg-resistant secondary calli were apparently propagated from *Agrobacterium* co-cultivated primary calli transformed with *ORF3*$^{\mathrm{HJX74}}$-*eGFP*, *ORF3*$^{\Delta100}$-*eGFP* and empty vector control (*eGFP*), whereas only some calli transformed with *ORF3-eGFP* grew. *ORF3* and *ORF3*$^{\mathrm{HJX74}}$ represent coding sequences of *ORF3* gene from E9 (with functional *ORF3*$^{\mathrm{Hap\text{-}1}}$) and HJX74 (with loss-of-function *ORF3*$^{\mathrm{Hap\text{-}2}}$) as shown in Supplementary Data 1, respectively, and *ORF3*$^{\Delta100}$ encodes ORF3 without N-terminal 100-aa containing mitochondrial targeting signal. Scale bars = 1 cm **(b)** Comparison of the selection frequency of Hyg-resistant callus. Data are shown as means ± SD (n = 3 biologically independent experiments). **(c)** Comparison of *eGFP* expression in Hyg-resistant calli. Data are shown as means ± SD (n = 3 and 5 biologically independent samples for *ORF3-eGFP* and others, respectively). Significant differences were determined by two-tailed Student’s t-tests (*p<0.05 and ***p<0.001 compared to *eGFP* control). Source data are provided as a Source Data file.

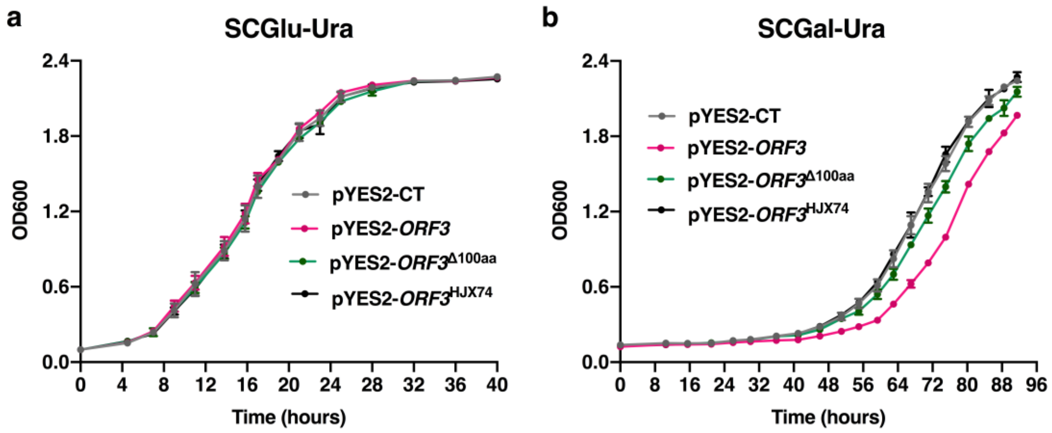

Supplementary Figure 14. Growth kinetic curves of *S. cerevisiae* INVSc1 transformants with or without ORF3 induction.

Exponential INVSc1 yeast cells transformed with a vector harboring *ORF3*, *ORF3*$^{\mathrm{HJX74}}$, *ORF3*$^{\Delta 100}$ or an empty vector (control, CT) were incubated at 30 °C in SCGlu-Ura growth medium **(a)** or SCGal-Ura induction medium **(b)**. All data are presented as means ± SD (n = 3 biologically independent clones). p-values was determined by two-tailed Student's t-tests. Source data are provided as a Source Data file.

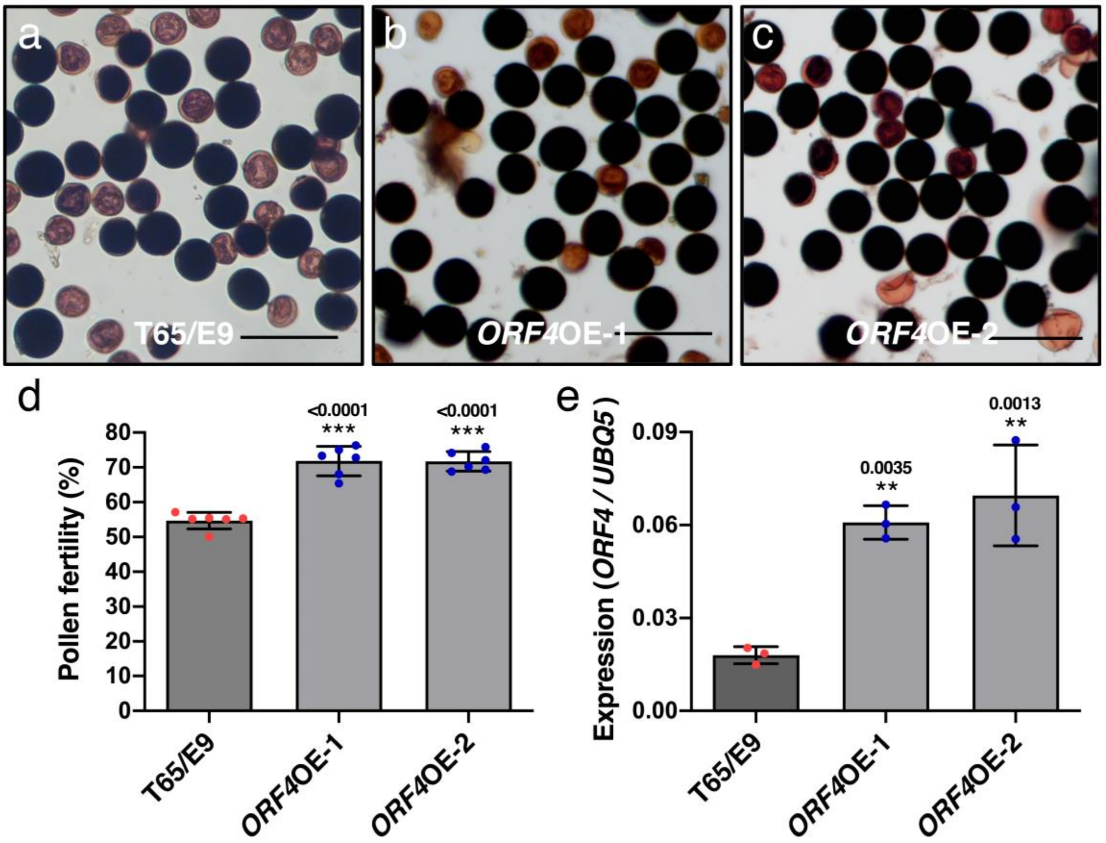

Supplementary Figure 15. Analysis of *ORF4* overexpression transgenic plants.

(a-c) I_2-KI stained pollen of a T65/E9 F_1 hybrid (a) and two T_0 transgenic *ORF4* overexpression plants (*ORF4*OE) with T65/E9 genotype (b-c). Scale bars = 100 µm. **(d)** Pollen fertility of T65/E9 F_1 hybrids and two T_0 *ORF4*OEs with T65/E9 genotype. Data are presented as means ± SD (*n* = 6 biologically independent samples). **(e)** *ORF4* expression in T65/E9 F_1 hybrids and two T_0 *ORF4*OEs with T65/E9 genotype. Data are presented as means ± SD (*n* = 3 biologically independent experiments). Significant differences were determined by two-tailed Student's *t*-tests (**p<0.01 and ***p<0.001 compared to T65/E9). Source data are provided as a Source Data file.

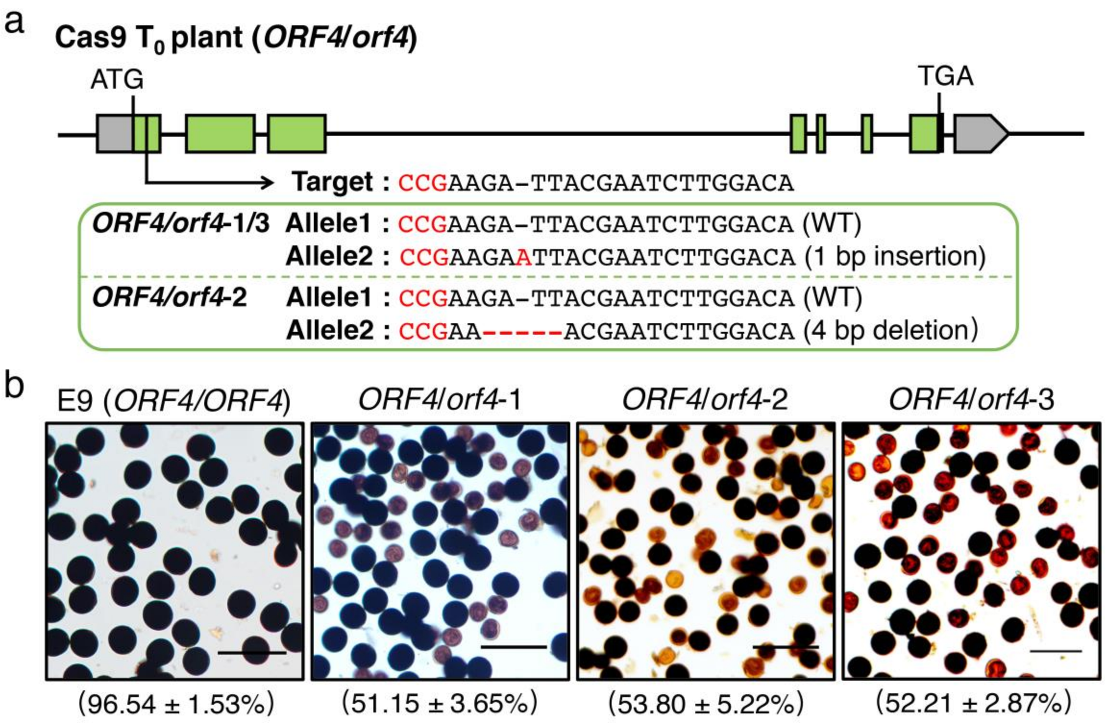

Supplementary Figure 16. Analysis of fertility of the hemizygous *orf4* mutants.

(a) Coding sequence of *ORF4* of E9 was targeted by CRISPR/Cas9, and three hemizygous *orf4* mutants were recovered. Target sequence and editing types are shown. PAM and insertion/deletion are highlighted in red. **(b)** I_2-KI stained pollen of E9 and T_0 hemizygous *orf4* mutants, and pollen fertility is shown as means ± SD below each type (*n* = 6 biologically independent samples for E9 and 3 biologically independent samples for others). *n* = 3 independent experiments. Scale bars = 100 μm. Source data are provided as a Source Data file.

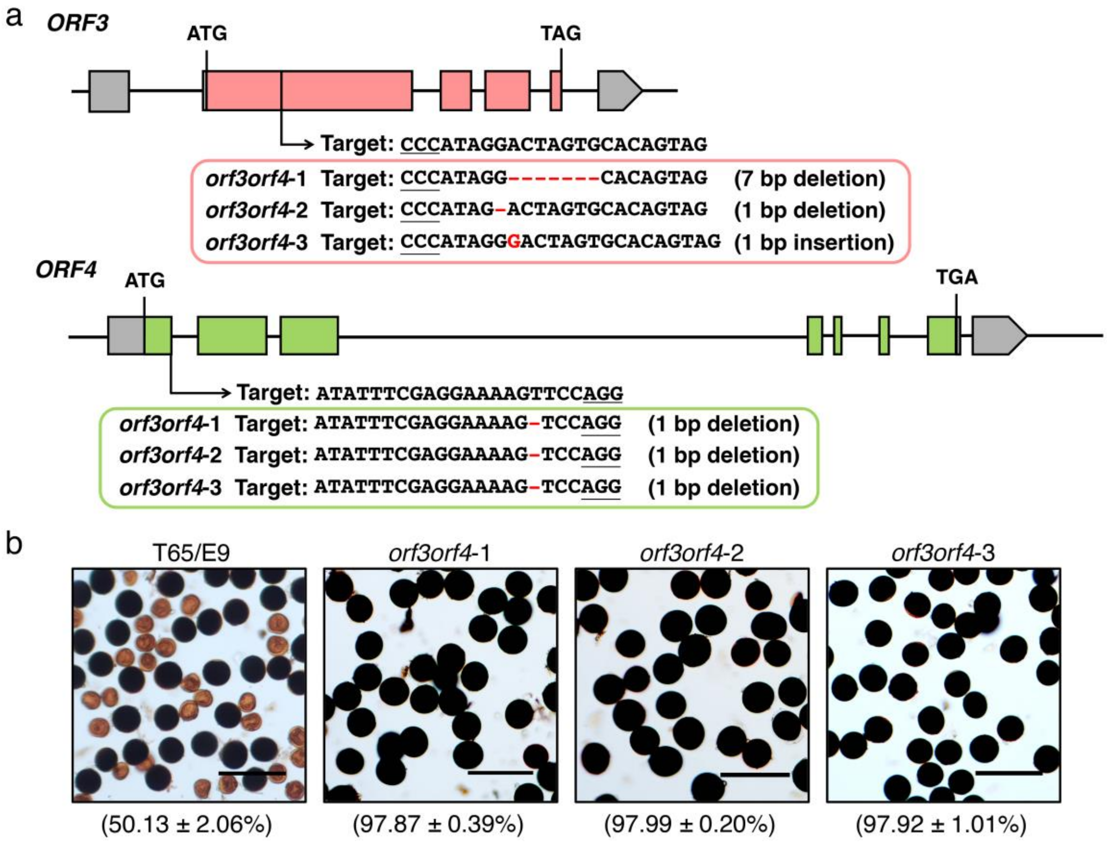

Supplementary Figure 17. Analysis of fertility of *orf3orf4* double mutants.

(a) Coding sequence of *ORF3* and *ORF4* were targeted by CRISPR/Cas9 simultaneously, and three homozygous *orf3orf4* mutants were recovered. Target sequence and editing types are shown. PAMs are underlined and Insertion/deletions are highlighted in red. **(b)** I_2-KI stained pollen of T65/E9 and three independent *orf3orf4* double mutants. Pollen fertility is shown as means ± SD below each type (n = 6 biologically independent samples for T65/E9 and 3 biologically independent samples for others). Scale bars = 100 μm. Source data are provided as a Source Data file.

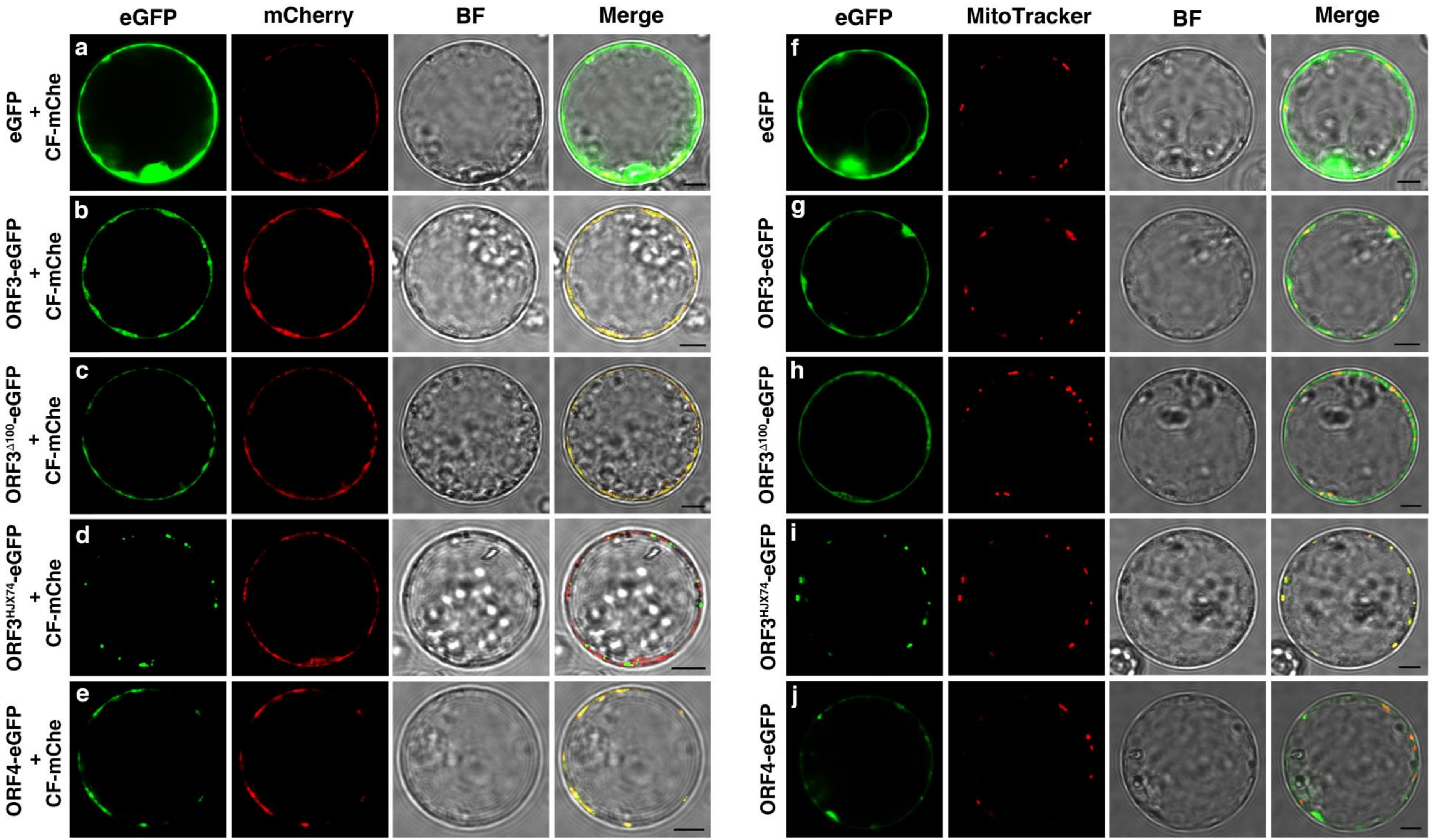
eGFP
mCherry
BF
Merge
eGFP
MitoTracker
BF
Merge
a
b
c
d
e
f
g
h
i
j
eGFP + CF-mChe
ORF3-eGFP + CF-mChe
ORF3Δ100-eGFP + CF-mChe
ORF3HJX74-eGFP + CF-mChe
ORF4-eGFP + CF-mChe
eGFP
ORF3-eGFP
ORF3Δ100-eGFP
ORF3HJX74-eGFP
ORF4-eGFP

Supplementary Figure 18. Subcellular localization of ORF3 and ORF4.

(a, f) eGFP expressed from an empty vector is localized in the cytosol and nucleus. **(b, g)** ORF3-eGFP co-localizes with a cytoplasmic foci marker (b) and mitochondrion-sepcific dye (MitoTracker Deep Red) (g). **(c, h)** Deletion of the putative mitochondrial targeting signal in ORF3$^{\Delta 100}$-eGFP affects its mitochondria localization (h) but not cytoplasmic foci localization (c). **(d, i)** ORF3^{HJX74}, which represents truncated ORF3 from HJX74 (with loss-of-function *ORF3*$^{Hap\text{-}2}$) as shown in Supplementary Data 1, shows localization signal only in mitochondria (i) but not in cytoplasmic foci (d). **(e, j)** ORF4-eGFP co-localizes with a cytoplasmic foci marker (e)but not mitochondrion-specific dye (j). n = 3 independent experiments. AtTZF1-mCherry was used as the cytoplasmic foci marker. CF, cytoplasmic foci; mChe, mCherry; BF, bright field. Scale bars = 5 μm.

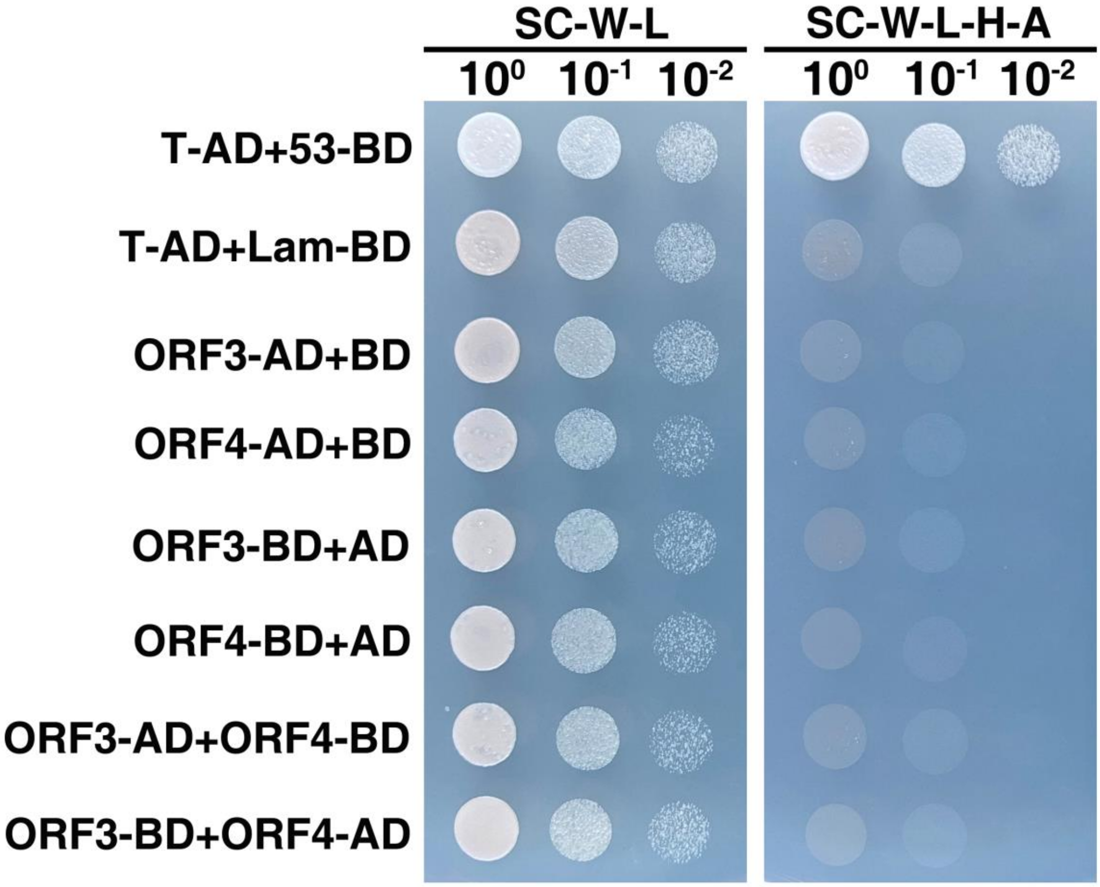

Supplementary Figure 19. Yeast two-hybrid assay test for interaction between ORF3 and ORF4.

Clones of yeast containing each combination of bait (BD) and prey (AD) vectors were grown on drop-out selection mediums without Trp and Leu (SC-W-L, left) or without Trp, Leu, His and Ade (SC-W-L-H-A, right), and no interaction between ORF3 and ORF4 could be detected. Interactions between T-AD and 53-BD was used as positive control, and interactions of T-AD + Lam-BD, ORF3-AD + BD, ORF4-AD + BD, AD + ORF3-BD, and AD + ORF4-BD were used as negative controls.

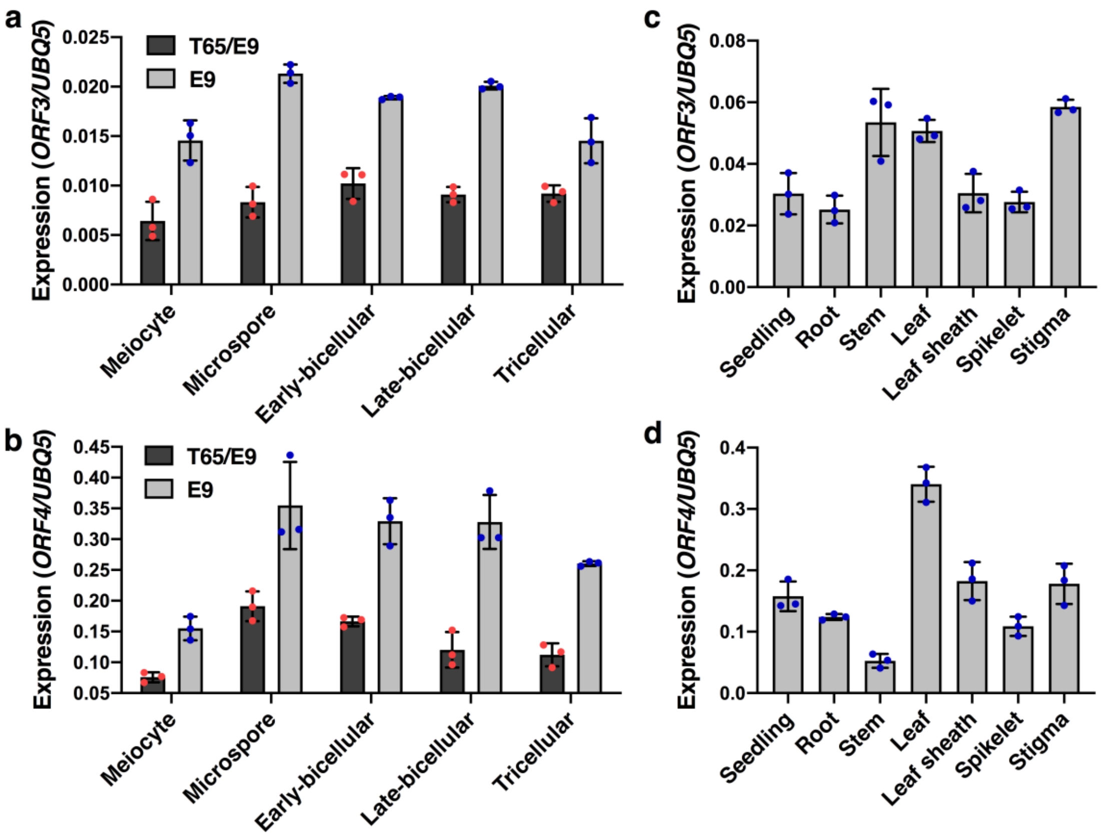

Supplementary Figure 20. Expression analysis of *ORF3* and *ORF4*.

(a-b) Expression of *ORF3* (a) and *ORF4* (b) in developing anthers of E9 and T65/E9 F_1 hybrids, analyzed by qRT-PCR. **(c-d)** Expression of *ORF3* (c) and *ORF4* (d) in different tissues of E9 plants. All data are presented as means ± SD (n = 3 biologically independent experiments). All primers are listed in Supplementary Data 4. Source data are provided as a Source Data file.

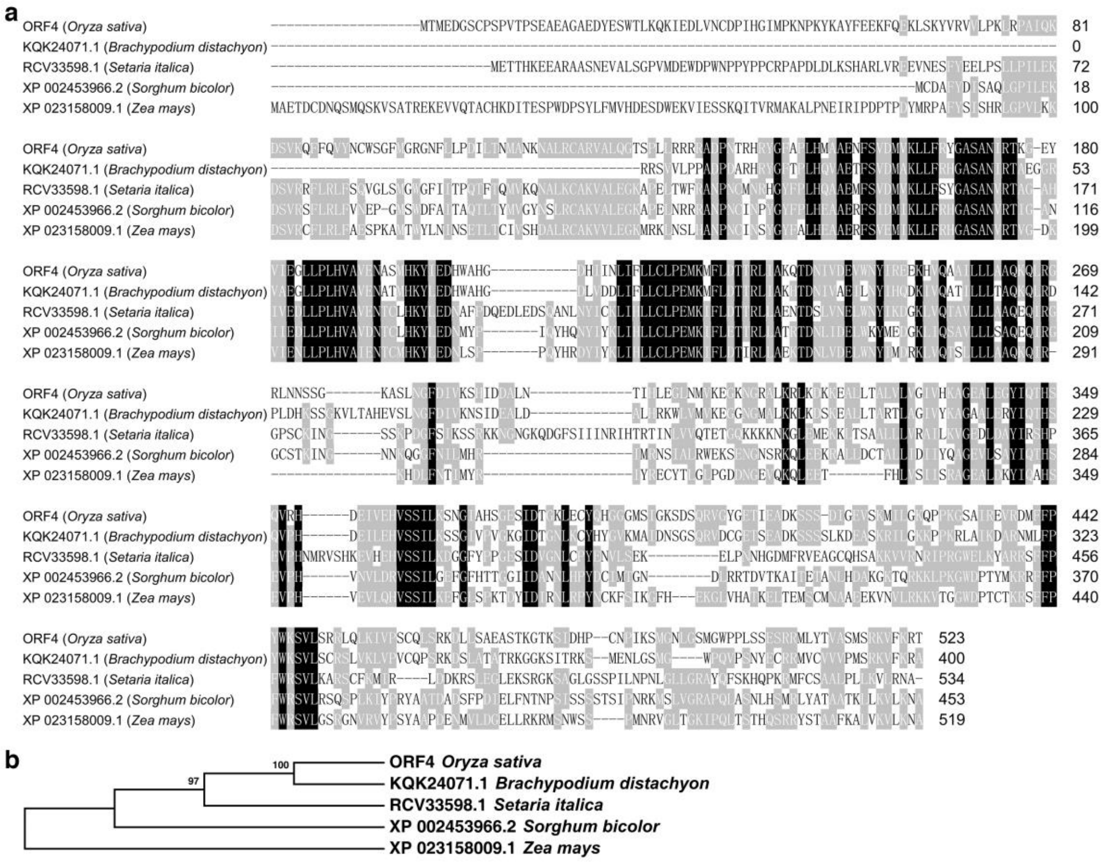

Supplementary Figure 21. Protein sequence alignments and phylogenetic tree of ORF4 homologs in *Poaceae*.

Protein sequence alignments **(a)** and phylogenetic tree **(b)** constructed using ORF4 and its best homologous sequences in *Brachypodium distachyon*, *Setaria italica*, *Sorghum bicolor*, and *Zea mays*. The phylogenetic tree was inferred using the Neighbor-Joining method. The bootstrap consensus tree inferred from 1,000 replicates is taken to represent the evolutionary history of the taxa analyzed.

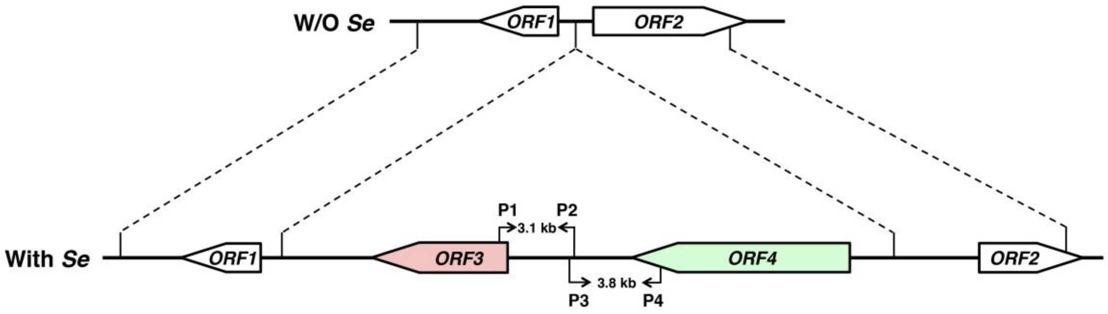

Supplementary Figure 22. Schematic illustration of PCR amplifying the PAV region.

Two pairs of primers were designed to amply the region spanning part of *ORF3* and *ORF4*, generating 3.1-kb and 3.8-kb fragments with 121-bp overlapping sequence in rice accessions with *Se*, while no PCR product could be obtained in rice accessions without *Se*. Then, specific primers in *ORF3* and *ORF4*, respectively, were further used to verify the presence or absence of *Se*. All primers are listed in Supplementary Data 4.

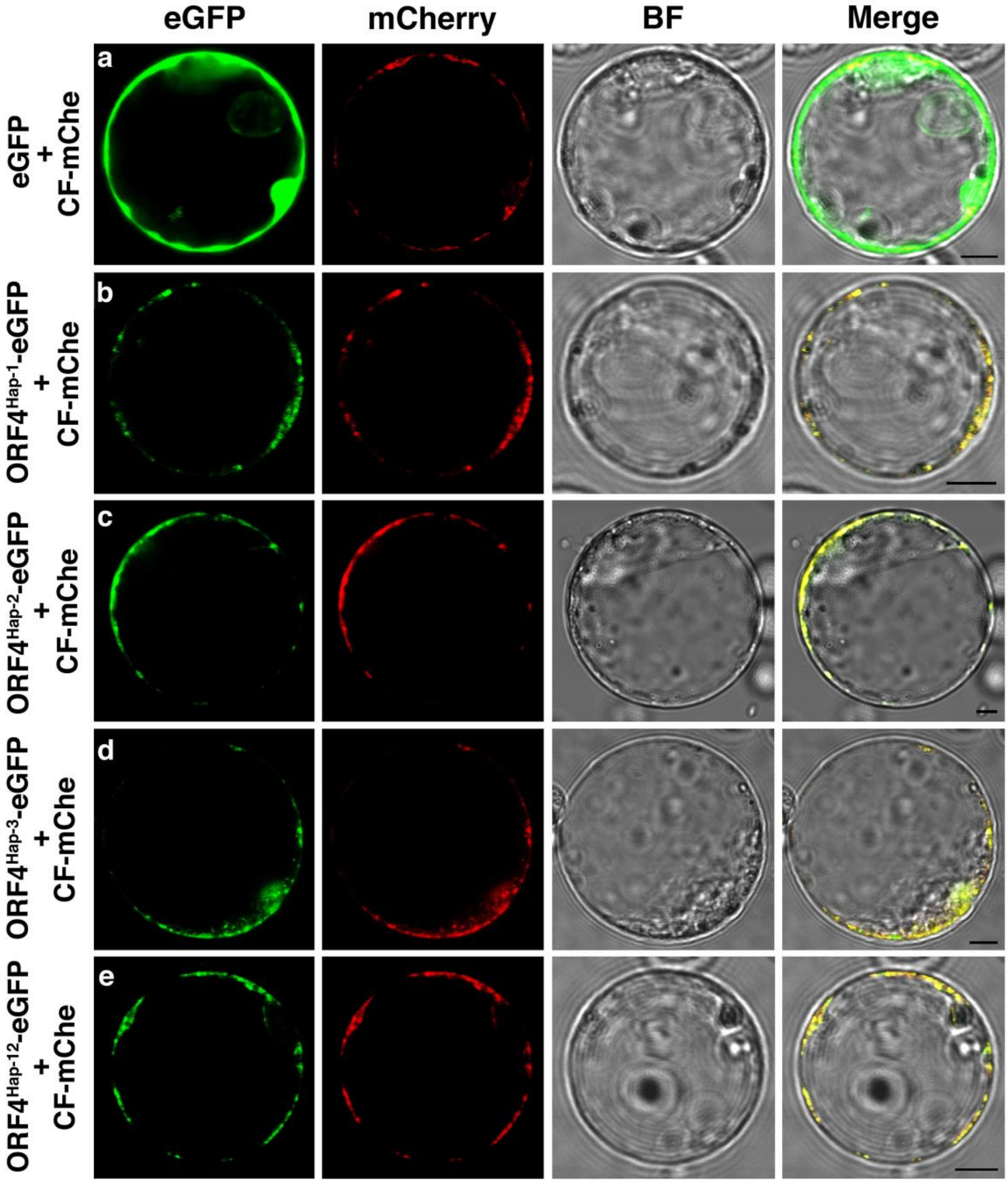

Supplementary Figure 23. Subcellular localization of different ORF4 haplotypes.

eGFP expressed from an empty vector is localized in the cytosol and nucleus **(a)**, while different ORF4 haplotypes (as shown in Supplementary Data 2) show similar localization in cytoplasmic foci **(b-e)**. *n* = 3 independent experiments. AtTZF1-mCherry was used as the cytoplasmic foci marker. CF, cytoplasmic foci; mChe, mCherry; BF, bright field. Scale bars = 5 μm.

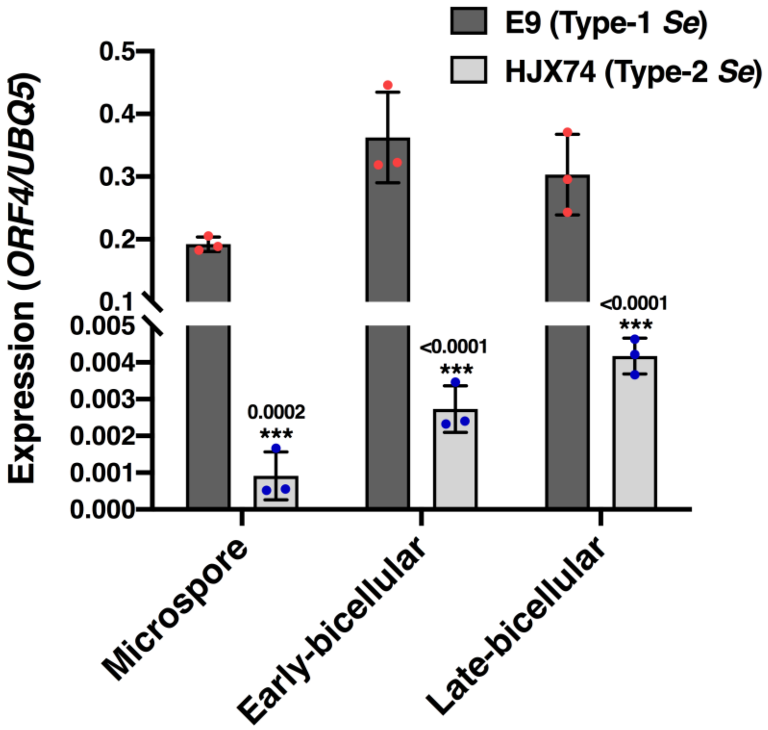

Supplementary Figure 24. A deletion in the *ORF4* promoter in Type-2 *Se* reduces *ORF4*'s expression

Expression of *ORF4* in the developing anthers of E9 (with *ORF4*$^{\text{Hap-1}}$ of Type-1 *Se*) and HJX74 (with *ORF4*$^{\text{Hap-3}}$ of Type-2 *Se*) by qRT-PCR. Type-2 *Se* contains a 369-bp deletion in the *ORF4* promoter. Data are presented as means ± SD (n = 3 biologically independent experiments). Significant differences were determined by two-tailed Student's t-tests (***p<0.001). Source data are provided as a Source Data file.

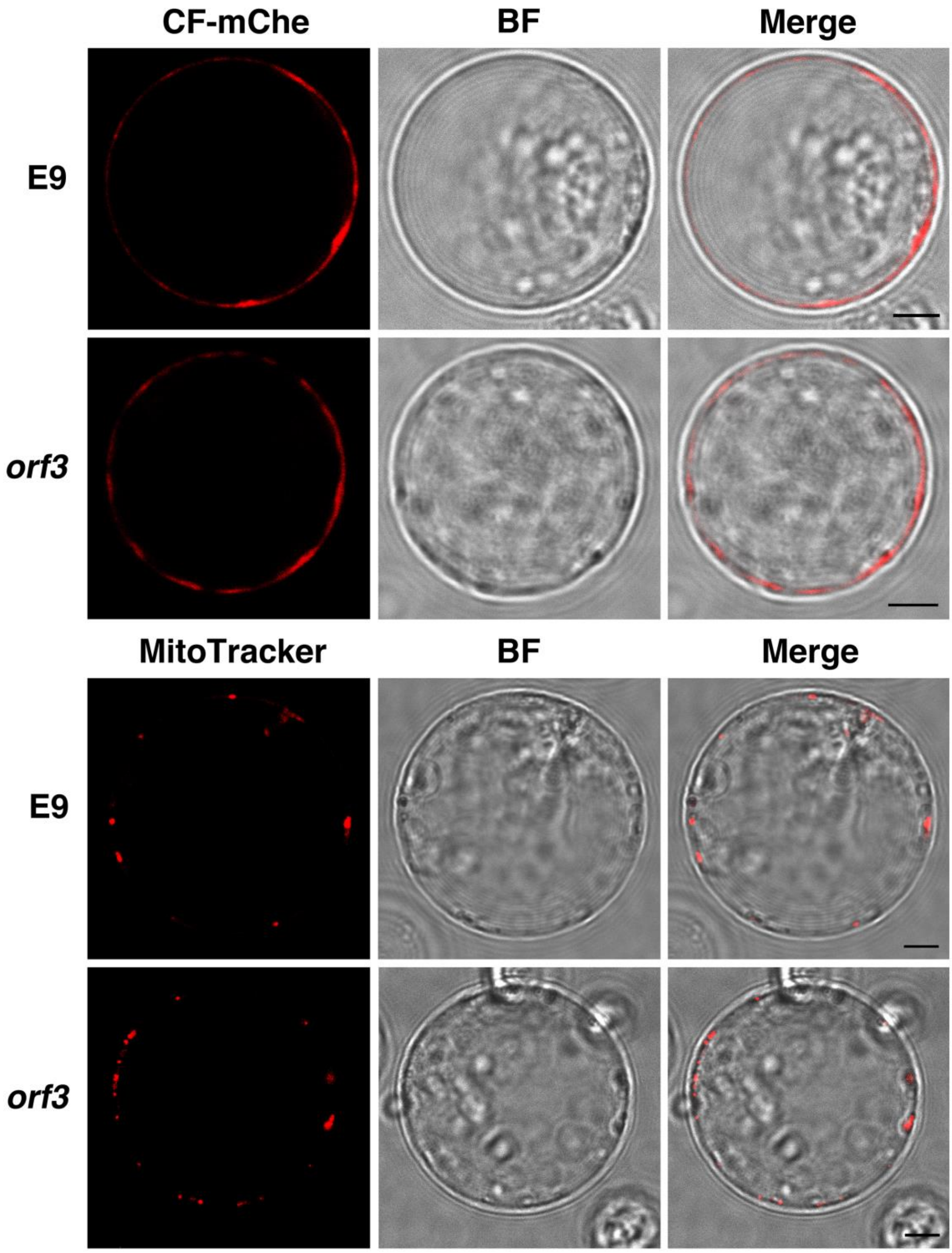

Supplementary Figure 25. Formation of Cytoplasmic foci and mitochondria in E9 and *orf3*.

Rice protoplasts extracted from E9 or *orf3-1* (as shown in Fig. 3) were transformed with the cytoplasmic foci marker (AtTZF1-mCherry) (upper two panels) or stained with mitochondrion-sepcific dye (MitoTracker Deep Red) (lower two panels). *n* = 3 independent experiments. CF, cytoplasmic foci; mChe, mCherry; BF, bright field. Scale bars = 5 μm.

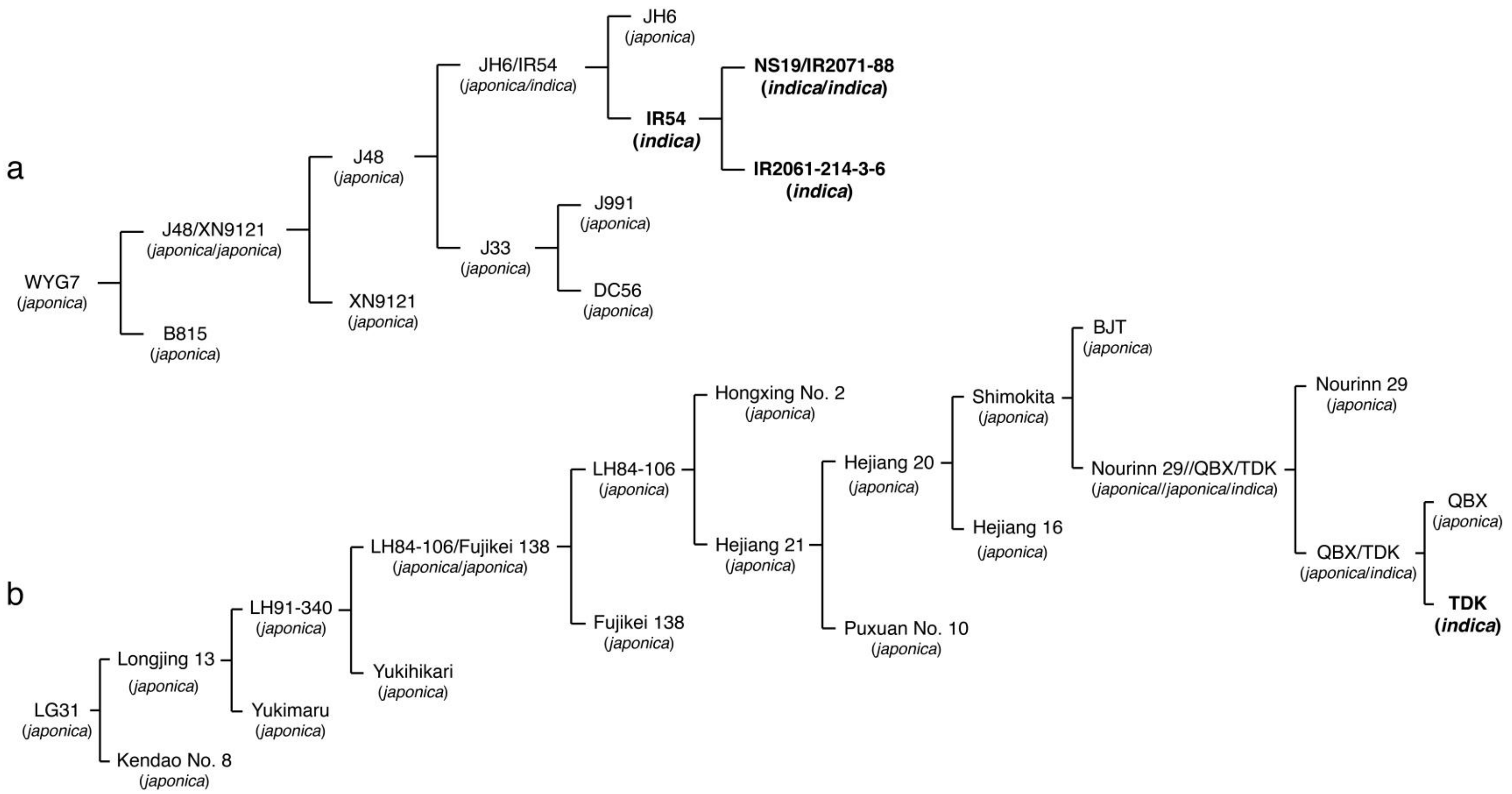

Supplementary Figure 26. Genealogical trees of *japonica* varieties WYG7 (a) and LG31 (b).

Indica varieties are highlighted in bold. Genealogical trees were deduced from the public available data on China Rice Data center (WYG7: https://www.ricedata.cn/variety/varis/601512.htm; LG31: https://www.ricedata.cn/variety/varis/609156.htm).

Supplementary Table 1. Segregation analysis of the BC_5F_2 population derived from crossing T65 with GLA4.

Chromosome	Molecular marker	No. of F_2 plants			χ^2(1:2:1)	*p* value
		T/T	*T/E*	*E/E*		
2	RM555	40	88	39	0.50	0.78
3	RM218	41	85	41	0.05	0.97
7	RM481	43	84	40	0.11	0.95
12	RM19	5	84	78	63.83	1.38E-14***
	RM453	5	89	73	56.10	6.57E-13***

Note: *T/T*, *E/E* and *T/E* designate homozygous genotypes for T65 and GLA4 and heterozygous genotype for T65/GLA4, respectively, at the corresponding molecular markers. *P* value was determined with a chi-square test analysis. *** represents significance at $p<0.001$.

Supplementary Table 2. Gametophytic transmission analysis using three genetic populations.

Crosses (♀/♂)	No. of plants (F_1)	Segregation *T/T*:*T/E*:*E/E*	χ^2(1:1)
T65/(*T/E*)	125	0:125:0	125.00
E9/(*T/E*)	218	0:0:218	218.00
(*T/E*)/E9	128	0:54:74	$3.13 < \chi^2_{0.05,1} = 3.84$

Note: *T/T*, *E/E* and *T/E* designate homozygous genotypes for T65 and E9 and heterozygous genotype for T65/E9, respectively.

Supplementary Table 3. Co-segregation of pollen fertility and tightly linked markers in key recombinants.

Recombinants	IND9	PSM559	IND37	PSM623	SNP6	IND51	IND48	IND52	PSM448	Pollen fertility
R-1	*T/T*	*T/T*	*T/E*	*T/E*	*T/E*	*T/E*	*T/E*	*T/E*	*T/E*	Semi-sterile
R-2	*T/E*	*T/E*	*E/E*	*E/E*	*E/E*	*E/E*	*E/E*	*E/E*	*E/E*	Fertile
R-3	*T/E*	*T/E*	*E/E*	*E/E*	*E/E*	*E/E*	*E/E*	*E/E*	*E/E*	Fertile
R-4	*T/E*	*T/E*	*T/E*	*E/E*	*E/E*	*E/E*	*E/E*	*E/E*	*E/E*	Fertile
R-5	*T/E*	*T/E*	*T/E*	*E/E*	*E/E*	*E/E*	*E/E*	*E/E*	*E/E*	Fertile
R-6	*T/E*	*T/E*	*T/E*	*T/E*	*E/E*	*E/E*	*E/E*	*E/E*	*E/E*	Fertile
R-7	*T/E*	*T/E*	*T/E*	*T/E*	*T/E*	*T/E*	*T/T*	*T/T*	*T/T*	Semi-sterile
R-8	*T/E*	*T/E*	*T/E*	*T/E*	*T/E*	*T/E*	*T/E*	*T/T*	*T/T*	Semi-sterile
R-9	*T/E*	*T/E*	*T/E*	*T/E*	*T/E*	*T/E*	*T/E*	*T/T*	*T/T*	Semi-sterile
R-10	*E/E*	*E/E*	*E/E*	*E/E*	*E/E*	*E/E*	*E/E*	*T/E*	*T/E*	Fertile
R-11	*E/E*	*E/E*	*E/E*	*E/E*	*E/E*	*E/E*	*E/E*	*T/E*	*T/E*	Fertile
R-12	*E/E*	*E/E*	*E/E*	*E/E*	*E/E*	*E/E*	*E/E*	*T/E*	*T/E*	Fertile

Note: *T/T*, *E/E* and *T/E* designate homozygous genotypes for T65 and E9 and heterozygous genotype for T65/E9, respectively, at the corresponding molecular markers.

Supplementary Table 4. Analysis of pollen fertility of the CRISPR/Cas9-engineered mutants of *orf1* and *orf2*.

Mutants	Targets	Editing types		Pollen fertility of mutants (%)	F_1	Pollen fertility of F_1 (%)
orf1^{T65}*-1*	*ORF1*^{T65}	Allele1:	AGAGGGGCGCATGCCTGC**C**GGCGG	97.40 ± 0.56	*orf1*^{T65}*-1*/E9	50.32 ± 2.08
		Allele2:	AGAGGGGCGCATGCCTGC**C**GGCGG			
		WT:	AGAGGGGCGCATGCCTGC-GGCGG			
orf1^{T65}*-2*	*ORF1*^{T65}	Allele1:	AGAGGGGCGCATGCCTG**A**CGGCGG	97.99 ± 0.19	*orf1*^{T65}*-2*/E9	42.70 ± 1.07
		Allele2:	AGAGGGGCGCATGCCTG**A**CGGCGG			
		WT:	AGAGGGGCGCATGCCTG-CGGCGG			
orf2^{T65}*-1*	*ORF2*^{T65}	Allele1:	AATATGATTAGGGC**--**TAGGTGG	97.92 ± 1.36	*orf2*^{T65}*-1*/E9	46.17 ± 1.79
		Allele2:	AATATGATTAGGGCA**---**GGTGG			
		WT:	AATATGATTAGGGCAATAGGTGG			
orf2^{T65}*-2*	*ORF2*^{T65}	Allele1:	AATATGATTAGGGCA-T-AGGTGG	98.68 ± 0.33	*orf2*^{T65}*-2*/E9	47.05 ± 3.05
		Allele2:	AATATGATTAGGGCAAT**T**AGGTGG			
		WT:	AATATGATTAGGGCAAT-AGGTGG			
orf1^{E9}*-1*	*ORF1*^{E9}	Allele1:	GGTCATTTGAAAACCAC**T**AGCAGG	96.42 ± 0.74	*orf1*^{E9}*-1*/T65	53.95 ± 2.89
		Allele2:	GGTCATTTGAAAACCAC**T**AGCAGG			
		WT:	GGTCATTTGAAAACCAC-AGCAGG			
orf1^{E9}*-2*	*ORF1*^{E9}	Allele1:	GGTCATTTGAAAA**-**CACAGCAGG	98.57 ± 1.05	*orf1*^{E9}*-2*/T65	52.73 ± 1.74
		Allele2:	GGTCATTTGAAAA**-**CACAGCAGG			
		WT:	GGTCATTTGAAAACCACAGCAGG			
orf1^{T65}*orf2*^{T65}*-1*	*ORF1*^{T65}	Allele1:	GGTCATTTGAAAACCAC**C**AGCAGG	97.52 ± 1.47	*orf1*^{T65}*orf2*^{T65}*-1*/E9	47.67 ± 2.78
		Allele2:	GGTCATTTGAAAACCAC**C**AGCAGG			

		WT:	GGTCATTTGAAAACCAC-AGCAGG			
	ORF2^{T65}	Allele1:	AGACTCTGCCATCACAA**-**CGCGG			
		Allele2:	AGACTCTGCCATCACAA**-**CGCGG			
		WT:	AGACTCTGCCATCACAAACGCGG			
orf1^{T65}*orf2*^{T65}*-2*	*ORF1*^{T65}	Allele1:	GGTCATTTGAA**-----**CAGCAGG	98.29 ± 0.67	*orf1*^{T65}*orf2*^{T65}*-2*/E9	50.25 ± 4.65
		Allele2:	GGTCATTTGAA**-----**CAGCAGG			
		WT:	GGTCATTTGAAAACCACAGCAGG			
	ORF2^{T65}	Allele1:	AGACTCTGCCATCAC**----**GCGG			
		Allele2:	AGACTCTGCCATCAC**----**GCGG			
		WT:	AGACTCTGCCATCACAAACGCGG			

Note: PAM is underlined, and mutations are highlighted in bold. Pollen fertility is shown as means ± SD (*n* = 3 biologically independent samples for mutants and 10 biologically independent samples for F_1s). Source data are provided as a Source Data file.

Supplementary Table 5. Pollen fertility of three independent *orf3* mutants.

Mutants	Targets	Editing types		Pollen fertility (%)
orf3-1	Target-1	GACA**T--------------**ACTAGTGCACAGTAG	(1 bp substitution and 16 bp deletion)	96.77 ± 1.13
	Target-2	TTAACTTCATGAACAGA**A**CGCCGG	(1 bp insertion)	
orf3-2	Target-1	GACAA**-------------**ACTAGTGCACAGTAG	(16 bp deletion)	93.01 ± 2.09
	Target-2	TTAACTTCATGAACAGACGCCGG	(WT)	
orf3-3	Target-1	AAACGAGCTTCCCATA**---…---**CGCCGG	(149 bp deletion)	94.92 ± 3.25
	Target-2	AAACGAGCTTCCCATA**---…---**CGCCGG	(included in the deletion of Target-1)	

Note: PAM is underlined, and nucleotide substitutions and deletions are highlighted in bold. Pollen fertility is shown as means ± SD (n = 3 biologically independent samples). Source data are provided as a Source Data file.

Supplementary Table 6. Pollen fertility of T_0 plants transformed with *ORF3* complementation or *ORF3* overexpression constructs in T65 background.

Constructs	PCR confirmation	Number of plants	Pollen fertility (%)	*p* value
*ORF3*CTL	positive	34	97.07 ± 2.21	0.50
	negative	7	97.70 ± 1.43	
*ORF3*OE	positive	3	97.50 ± 1.93	0.18
	negative	25	98.50 ± 0.95	

Note: Pollen fertility is shown as means ± SD (n = 15, 7, 3, and 15 biologically independent samples for *ORF3*CTL-positive, *ORF3*CTL-negative, *ORF3*OE-positive and *ORF3*OE-negative, respectively). *P* value represents two-tailed Student's *t*-tests comparing the pollen fertility of transgenic plants that contain a given transgene with that of recovered plants lacking the transgene. *ORF3*CTL and *ORF3*OE represent *ORF3* complementation and overexpression constructs, respectively. Source data are provided as a Source Data file.

Supplementary Table 7. Analysis of pollen fertility of F_1 progeny from crosses of complementation transgenic lines with E9.

Crosses (♀/♂)	Genotype	Pollen fertility (%)	*p* value
T65/E9	*T/E*	50.99 ± 3.43	-
*ORF4*CTL-1/E9	*T/E; ORF4/-*	74.75 ± 8.86	5.13E-06***
*ORF4*CTL-2/E9	*T/E; ORF4/-*	73.08 ± 1.13	8.43E-10***
*ORF4*CTL-3/E9	*T/E; ORF4/-*	73.28 ± 0.10	6.99E-09***
ORF1^{E9}CTL-1/E9	*T/E*; *ORF1*^{E9}/-	49.10 ± 1.49	0.13
ORF1^{E9}CTL-2/E9	*T/E*; *ORF1*^{E9}/-	51.08 ± 1.12	0.94
ORF1^{E9}CTL-3/E9	*T/E*; *ORF1*^{E9}/-	50.65 ± 3.06	0.82
ORF2^{E9}CTL-1/E9	*T/E*; *ORF2*^{E9}/-	48.27 ± 2.46	0.06
ORF2^{E9}CTL-2/E9	*T/E*; *ORF2*^{E9}/-	51.04 ± 1.25	0.97
ORF2^{E9}CTL-3/E9	*T/E*; *ORF2*^{E9}/-	50.90 ± 5.91	0.97

Note: Pollen fertility is shown as means ± SD (n = 10 biologically independent samples). *P* value represents two-tailed Student's *t*-tests comparing the pollen fertility of plants containing a given transgene with that of plants lacking the transgene in a heterozygous *T/E* background. *** represent significance at p<0.01 and p<0.001, respectively. Source data are provided as a Source Data file.

Supplementary Table 8. *ORF4* transgene rescues pollen fertility of the *T/E* genotype plants.

Transgenic lines	T_1 genotype	Pollen fertility (%)	Expected ratio	Number of plants	Expected value	χ^2 value
*ORF4*CTL-1	*T/T; ORF4/-* and *T/T; ORF4/ORF4*	96.65 ± 2.23	2/12	34	40	$1.11 < \chi^2_{0.05,5} = 11.07$
	T/E; ORF4/-	79.07 ± 2.87	3/12	62	60	
	T/E; ORF4/ORF4	96.50 ± 2.48	2/12	41	40	
	T/E; -/-	53.51 ± 3.70	1/12	21	20	
	E/E; ORF4/- and *E/E; ORF4/ORF4*	98.02 ± 1.79	3/12	62	60	
	E/E; -/-	96.23 ± 1.62	1/12	20	20	
*ORF4*CTL-2	*T/T; ORF4/-* and *T/T; ORF4/ORF4*	98.07 ± 0.90	2/12	24	33.5	$4.24 < \chi^2_{0.05,5} = 11.07$
	T/E; ORF4/-	73.87 ± 0.92	3/12	49	50.25	
	T/E; ORF4/ORF4	98.03 ± 1.39	2/12	35	33.5	
	T/E; -/-	48.99 ± 5.28	1/12	21	16.75	
	E/E; ORF4/- and *E/E; ORF4/ORF4*	97.87 ± 1.54	3/12	54	50.25	
	E/E; -/-	97.37 ± 1.75	1/12	18	16.75	

Note: *T/T*, *E/E* and *T/E* designate homozygous genotypes for T65 and E9 and heterozygous genotype for T65/E9, respectively. *ORF4* represents the complementation transgene construct of *ORF4*. Pollen fertility is shown as means ± SD (n = 8 biologically independent samples). Source data are provided as a Source Data file.

Supplementary Table 9. Segregation of genotypes in progeny of a T65/E9 F_1 hybrid and two T_0 *ORF4*OEs with T65/E9 genotype.

Plants	No. of plants	Segregation *T/T*:*T/E*:*E/E*	*T/T* plants (%)
T65/E9	316	0:140:176	0
*ORF4*OE-1	308	43:153:112	13.96
*ORF4*OE-2	157	25:62:70	15.92

Note: *T/T*, *E/E* and *T/E* designate homozygous genotypes for T65 and E9 and heterozygous genotype for T65/E9, respectively.

Supplementary Table 10. Analysis of pollen fertility of CRISPR/Cas9-engineered *orf4* mutants in T65/E9 and E9 backgrounds.

Experiment	Target	Background	Genotype	Number of plants	Pollen fertility (%)
Exp-1	Target-1	T65/E9	*ORF4*/-	17	49.05 ± 1.87
			orf4/-	0	-
		E9	*ORF4*/*ORF4*	33	97.88 ± 0.59
			ORF4/*orf4*-1	1	51.15 ± 3.65
			orf4/*orf4*	0	-
Exp-2	Target-1	E9	*ORF4*/*ORF4*	20	96.54 ± 1.53
			ORF4/*orf4*-2/3	2	52.95 ± 3.77
			orf4/*orf4*	0	-
Exp-3	Target-2	E9	*ORF4*/*ORF4*	12	97.35 ± 1.03
			ORF4/*orf4*	0	-
			orf4/*orf4*	0	-
Exp-4	Target-3	E9	*ORF4*/*ORF4*	10	96.85 ± 1.89
			ORF4/*orf4*	0	-
			orf4/*orf4*	0	-

Note: *orf4* indicates the CRISPR/Cas9-engineered *ORF4* allele. Pollen fertility is shown as means ± SD (n = 10, 15, 3, 10, 2, 8, and 8 biologically independent samples for *ORF4/-* (Exp-1), *ORF4/ORF4* (Exp-1), *ORF4/orf4*-1 (Exp-1), *ORF4/ORF4* (Exp-2), *ORF4/orf4*-2/3 (Exp-2), *ORF4/ORF4* (Exp-3) and *ORF4/ORF4* (Exp-4), respectively). Source data are provided as a Source Data file.

Supplementary Table 11. Segregation of genotypes in the T_1 progeny of the hemizygous *orf4* mutants.

T_0 genotype	T_1 genotype	Editing type	No. of plants	χ^2(1:2:1)
ORF4/orf4-1	*ORF4/ORF4*	Allele1: WT<br>Allele2: WT	21	20.14 > $\chi^2_{0.01,2}$ =9.21
	ORF4/orf4-1	Allele1: WT<br>Allele2: 1 bp insertion	23	
	orf4-1/*orf4*-1	Allele1: 1 bp insertion<br>Allele2: 1 bp insertion	0	
ORF4/orf4-2	*ORF4/ORF4*	Allele1: WT<br>Allele2: WT	14	13.40 > $\chi^2_{0.01,2}$ =9.21
	ORF4/orf4-2	Allele1: WT<br>Allele2: 4 bp deletion	26	
	orf4-2/*orf4*-2	Allele1: 4 bp deletion<br>Allele2: 4 bp deletion	0	

Supplementary Table 12. Frequency of absence of the PAV region in different species.

Species	Groups	Number of accessions		Frequency of absence (%)
O. punctata	BB-genome	absence of the PAV region	4	100
		presence of the PAV region	0	
O. eichingeri	CC-genome	absence of the PAV region	2	100
		presence of the PAV region	0	
O. officinalis	CC-genome	absence of the PAV region	3	100
		presence of the PAV region	0	
O. australiensis	EE-genome	absence of the PAV region	3	100
		presence of the PAV region	0	
O. brachyantha	FF-genome	absence of the PAV region	2	100
		presence of the PAV region	0	
O. meyeriana	GG-genome	absence of the PAV region	3	100
		presence of the PAV region	0	
O. longistaminata	AA-genome	absence of the PAV region	28	100
		presence of the PAV region	0	
O. glumaepatula	AA-genome	absence of the PAV region	16	100
		presence of the PAV region	0	
O. meridionalis	AA-genome	absence of the PAV region	0	0
		presence of the PAV region	5	
O. barthii	AA-genome	absence of the PAV region	0	0
		presence of the PAV region	4	
O. rufipogon	AA-genome	absence of the PAV region	235	68.71
		presence of the PAV region	107	
O. nivara	AA-genome	absence of the PAV region	34	77.27
		presence of the PAV region	10	
O. glaberrima	AA-genome	absence of the PAV region	0	0
		presence of the PAV region	7	
indica	AA-genome	absence of the PAV region	22	9.32
		presence of the PAV region	214	
japonica	AA-genome	absence of the PAV region	132	89.19
		presence of the PAV region	16	

Supplementary Table 13. Frequency of PAV types in all rice accessions with the PAV region.

PAV types	Species	Number of accessions	Frequency of type in rice accessions with the PAV region (%)
Type-1	*O. meridionalis*	0	27.55
	O. barthii	0	
	O. rufipogon	19	
	O. nivara	1	
	O. glaberrima	0	
	indica	74	
	japonica	6	
Type-2	*O. meridionalis*	0	64.19
	O. barthii	0	
	O. rufipogon	78	
	O. nivara	6	
	O. glaberrima	0	
	indica	139	
	japonica	10	
Type-3	*O. meridionalis*	0	3.86
	O. barthii	0	
	O. rufipogon	10	
	O. nivara	3	
	O. glaberrima	0	
	indica	1	
	japonica	0	
Type-4	*O. meridionalis*	0	3.03
	O. barthii	4	
	O. rufipogon	0	
	O. nivara	0	
	O. glaberrima	7	
	indica	0	
	japonica	0	
Type-5	*O. meridionalis*	5	1.38
	O. barthii	0	
	O. rufipogon	0	
	O. nivara	0	
	O. glaberrima	0	
	indica	0	
	japonica	0	

Supplementary Table 14. Frequency of PAV types in different species with the PAV region.

Species	PAV types	Number of accessions	Frequency of PAV types in species with the PAV region (%)
O. meridionalis	Type-1	0	0
	Type-2	0	0
	Type-3	0	0
	Type-4	0	0
	Type-5	5	100
O. barthii	Type-1	0	0
	Type-2	0	0
	Type-3	0	0
	Type-4	4	100
	Type-5	0	0
O. rufipogon	Type-1	19	17.76
	Type-2	78	72.90
	Type-3	10	9.35
	Type-4	0	0
	Type-5	0	0
O. nivara	Type-1	1	10.00
	Type-2	6	60.00
	Type-3	3	30.00
	Type-4	0	0
	Type-5	0	0
O. glaberrima	Type-1	0	0
	Type-2	0	0
	Type-3	0	0
	Type-4	7	100
	Type-5	0	0
indica	Type-1	74	34.58
	Type-2	139	64.95
	Type-3	1	0.47
	Type-4	0	0
	Type-5	0	0
japonica	Type-1	6	37.50
	Type-2	10	62.50
	Type-3	0	0
	Type-4	0	0
	Type-5	0	0
