# Supplementary material for: Two complementary genes in a presence-absence variation contribute to indica-japonica reproductive isolation in rice
Source: Nat Commun. 2023 Jul 28;14:4531. doi: 10.1038/s41467-023-40189-x (PMC10382596; doi:10.1038/s41467-023-40189-x)
Supplement: Supplementary file 3 — Description of Additional Supplementary Files [file 41467_2023_40189_MOESM3_ESM.pdf]

Description of Additional Supplementary Files

File Name: Supplementary Data 1

Description: Haplotype analysis of ORF3 in *Oryza* species

File Name: Supplementary Data 2

Description: Haplotype analysis of ORF4 in *Oryza* species

File Name: Supplementary Data 3

Description: *Se* haplotypes in *Oryza* species

File Name: Supplementary Data 4

Description: Primers used in this research

File Name: Supplementary Data 5

Description: Rice accessions used for haplotype survey and evolutionary analysis
